# Evidence supporting a critical contribution of intrinsically disordered regions to the biochemical behavior of full-length human HP1γ

**DOI:** 10.1007/s00894-015-2874-z

**Published:** 2015-12-17

**Authors:** Gabriel Velez, Marisa Lin, Trace Christensen, William A. Faubion, Gwen Lomberk, Raul Urrutia

**Affiliations:** Laboratory of Epigenetics and Chromatin Dynamics, Gastroenterology Research Unit, Department of Biochemistry and Molecular Biology, Mayo Clinic, 200 First Street SW, Guggenheim 10, Rochester, MN 55905 USA; Laboratory of Epigenetics and Chromatin Dynamics, Gastroenterology Research Unit, Department of Biophysics, Mayo Clinic, Rochester, MN USA; Laboratory of Epigenetics and Chromatin Dynamics, Gastroenterology Research Unit, Department of Medicine, Mayo Clinic, Rochester, MN USA; Medical Scientist Training Program, University of Iowa, Iowa City, IA USA

**Keywords:** HP1, HP1γ, CBX3, Molecular modeling, Molecular dynamics, Epigenetics, Chromatin

## Abstract

HP1γ, a non-histone chromatin protein, has elicited significant attention because of its role in gene silencing, elongation, splicing, DNA repair, cell growth, differentiation, and many other cancer-associated processes, including therapy resistance. These characteristics make it an ideal target for developing small drugs for both mechanistic experimentation and potential therapies. While high-resolution structures of the two globular regions of HP1γ, the chromo- and chromoshadow domains, have been solved, little is currently known about the conformational behavior of the full-length protein. Consequently, in the current study, we use threading, homology-based molecular modeling, molecular mechanics calculations, and molecular dynamics simulations to develop models that allow us to infer properties of full-length HP1γ at an atomic resolution level. HP1γ appears as an elongated molecule in which three Intrinsically Disordered Regions (IDRs, 1, 2, and 3) endow this protein with dynamic flexibility, intermolecular recognition properties, and the ability to integrate signals from various intracellular pathways. Our modeling also suggests that the dynamic flexibility imparted to HP1γ by the three IDRs is important for linking nucleosomes with PXVXL motif-containing proteins, in a chromatin environment. The importance of the IDRs in intermolecular recognition is illustrated by the building and study of both IDR2 HP1γ−importin-α and IDR1 and IDR2 HP1γ−DNA complexes. The ability of the three IDRs for integrating cell signals is demonstrated by combined linear motif analyses and molecular dynamics simulations showing that posttranslational modifications can generate a histone mimetic sequence within the IDR2 of HP1γ, which when bound by the chromodomain can lead to an autoinhibited state. Combined, these data underscore the importance of IDRs 1, 2, and 3 in defining the structural and dynamic properties of HP1γ, discoveries that have both mechanistic and potentially biomedical relevance.

**Electronic supplementary material:**

The online version of this article (doi:10.1007/s00894-015-2874-z) contains supplementary material, which is available to authorized users.

## Introduction

The heterochromatin protein 1 (HP1) family of histone mark readers, the focus of the current study, was one of the first types of chromatin regulators to be identified [1, 2]. This family of proteins participates in evolutionarily conserved processes in organisms ranging from early eukaryotes to humans [2, 3]. Human cells produce three different HP1 protein isoforms, HP1α (CBX5), HP1β (CBX1), and HP1γ (CBX3), which regulate the expression of entire networks of genes that are critical for normal embryonic development and the maintenance of most homeostatic processes, including cell cycle control, proliferation, apoptosis, differentiation, and DNA damage response [2, 4]. In addition, the expression and deregulation of HP1-mediated processes associate with the development, spreading, and prognosis of several cancers [4]. Consequently, better understanding of the biochemical properties of HP1 proteins has both biological and medical implications.

The current work represents an extension of work in our laboratory, which seeks to understand the biological and pathobiological roles of HP1γ. Early biochemical studies revealed that HP1γ recognizes and binds specific di- and tri-methylated forms of histones (K9H3 and K26H1) and translates this biochemical information into a defined pattern of gene expression [5–7]. The ability of HP1γ to recognize this mark was subsequently mapped to a small region within the N-terminal domain, known as chromodomain [8]. In addition, HP1γ uses this chromodomain to recruit the related histone methyltransferases, G9a and GLP, which write dimethylated K9 histone marks as part of a positive-feedback loop that leads to increased concentration of reader–writer complexes on specific genomic regions where they are needed to regulate gene expression [3]. G9a and GLP have the ability to auto-methylate at an internal K-containing peptide, which mimics methylated-histones (histone mimicry) [9]. HP1γ also recruits an additional histone methyltransferase protein, SUV39H1, in a manner that is independent of its methylation status, but rather contains a specific linear motif with a PXVXL consensus sequence [10]. For recognizing and binding the PXVXL motif, HP1γ must first form homodimers or heterodimers with HP1α or HP1β [3, 10]. Dimerization and PXVXL recognition, which is imparted to HP1γ by its N-terminal chromoshadow domain, recruits additional chromatin regulators that may impart further instructions for the regulation of genomic and epigenomic functions [3, 10]. Thus, due to the functional importance of both the chromo- and chromoshadow domains, structural studies have begun to focus on deciphering the biophysical properties that determine their function, in the hope that this knowledge may aid in the development of drugs for manipulating HP1γ-mediated processes in experimental and therapeutic settings [3].

Several laboratories have focused on studying the function of less well-characterized regions of the HP1γ molecules, namely the most N- and C-terminal regions located between the chromo and chromoshadow domains. Unfortunately, in this regard, no NMR or X-ray crystallographic studies have yet yielded any useful information regarding the properties of these less-known domains [11, 12]. Therefore, there is a need for a better understanding of the structure and biophysical behavior of full-length human HP1γ by assigning biophysical properties of those domains for which data at the atomic resolution is lacking, establishing their role in molecular connectivity and flexibility as well as intermolecular interactions. Consequently, using a combination of structural bioinformatics, molecular modeling methods, and molecular dynamics approaches, we here report that HP1γ is an elongated molecule, in which three Intrinsically Disordered Regions (IDRs, 1, 2, and 3) endow this protein with dynamic flexibility, intermolecular recognition properties, and the ability to integrate signals from various intracellular pathways. Our models and the inferences derived from them integrate, complement, explain, and extend available experimental data, providing new insights that can serve as the structural rationale for future experimentations and drug design.

## Materials and methods

### Generation of a structural model for full-length HP1γ

**Fig. 1:**
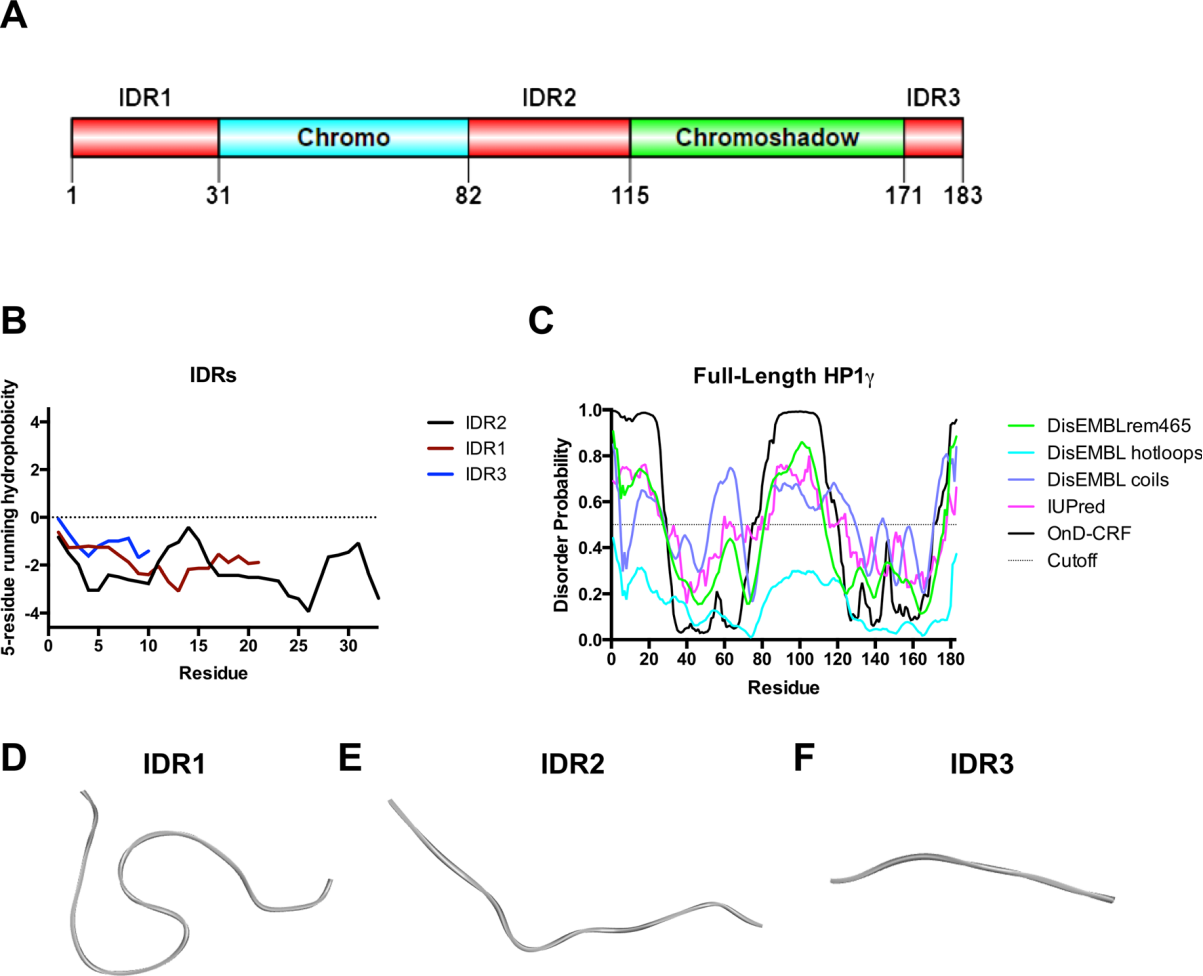
Structural bioinformatics reveal the HP1γ IDR regions to have a high propensity toward disorder. **a** Linear domain graph showing amino acid positions for IDR1 (Met1 – Val31), IDR2 (Ala82 – Arg115), and IDR3 (Arg171 – Gln183). **b** Hydrophobicity plot reveals the IDRs have a high polar to hydrophobic ratio of residues, a feature that characterizes intrinsically disordered proteins. **c** Disorder meta-prediction for full-length HP1γ reveals the IDRs to have a higher propensity toward disorder, while the chromo and chromoshadow domain are predicted to be ordered regions of the protein. Disorder probability values above the cut-off value of 0.5 are considered to be disordered. **d** Structure predictions of IDR1 **e**, IDR2, and IDR3 **f** predicted by threading algorithms show that threading cannot model these regions in any secondary or tertiary conformation

To evaluate the disorder probability of the N- (Met1 – Val31) and C-terminal (Arg171 – Gln183) regions as well as the HP1γ linker (Ala82 – Arg115), we used a meta-prediction approach that integrated the data from PrDOS [13], metaPrDOS [14], POODLE [15], DISpro [16], DisEMBL [17], IUPred [18], PONDR-FIT [19], PreDisorder [20], OnD-CRF [21], RONN [22], FoldIndex [23], DISOclust [24], and GlobPlot2 [25]. As a negative control, we subjected the sequence of an ordered alpha helix from HP1γ (PQIVIAFYEER; residues 161–171) to the same analysis. The N-terminal domain of the HP1γ isoforms were modeled using the threading algorithms LOMETS [26] and MUSTER [27]. The C-terminal regions were modeled as disordered regions using homology-based modeling with the mouse structure of HP1β (PDB: 3Q6S) [28]. In addition, a final homology-based model of the HP1γ linker regions were built using the solved structure of the bipartite NLS from nucleophosmin (PDB: 1PJN) in MODELLER [28]. All of these models congruently predict that these HP1γ sequences constitute Intrinsically Disorder Regions, herein called IDR1, IDR2, and IDR3. Full models of the HP1 isoforms (PDB: 1GUW and 3Q6S for HP1β; 3KUP and 3DM1 for HP1γ; as well as 3I3C and 3FDT for HP1α) were constructed using the peptide bonding function of the Builder feature of Discovery Studio 4.1 [29] to establish the following molecular connectivity IDR1-chromodomain-IDR2-chromoshadow domain-IDR3 (Fig. 1a). Biophysical properties of this model (i.e., volume, electrostatics, etc.) were calculated using VADAR [30] and the 3 V Volume Calculator [31].

### Modeling of HP1 complexes

The HP1γ−HP1γ homodimer and heterodimers with HP1α and HP1β were docked by homology using the structure of the chromoshadow domain of the mouse HP1α and HP1β (PDB: 1GUW and 3Q6S for HP1β; 3KUP and 3DM1 for HP1γ; as well as 3I3C and 3FDT for HP1α). The three-dimensional complex structure of HP1γ bound with α-importin was generated by docking its linker region to a previously solved structure of α-importin (PDB: 1PJN) to achieve maximal intermolecular interactions by the bipartite cluster of basic amino acids as previously described [32]. For this purpose, the IDR2 region was modeled first by homology to the conformation described for the isolated N1N2 NLS (PDB: 1PJN), which is a paradigm for docking homologous peptides to α−importin. Because of its high level of structural similarity (RMSD = 0.3), this peptide was easily docked manually to the respective NLS receptor of α−importin. Intermolecular interactions of the HP1γ-α-importin complex, including salt bridge interactions, hydrogen bonds, electrostatic interactions, and hydrophobic interactions, were calculated in the Receptor-Ligand function of Discovery Studio Client 4.1 using the default parameters [29]. The three-dimensional complex structure of HP1γ bound to B-DNA was generated by using DP-Dock [33], which has been well validated by our laboratory and others [34]. DP-Dock uses a nonspecific B-DNA model to probe the binding site on a 3D model of a protein that is known to bind DNA, but for which the specific contacts are unknown. Using the structure of a DNA binding protein as input, the method first automatically generated an ensemble of protein–DNA complexes obtained by rigid-body docking with nonspecific canonical B-DNA molecules [33]. Models were subsequently selected by clustering and ranking them according to their DNA–protein interfacial energies [33].

### Molecular dynamics (MD) simulations

The MD simulations of HP1γ and its complexes were performed using the all-atom force field in CHARMm c36b2 at a temperature of 300 K (NVT ensemble) [35]. The molecule was first energy-minimized using a two-step protocol of steepest descent and conjugated gradients. All of these steps were done using the SHAKE procedure [36]. A distance-dependent dielectrics implicit solvent model was used with a dielectric constant of 80 and a pH of 7.4. Using the same procedure, additional MD simulations were performed on models of HP1 complexes and on HP1γ mutants. In order to better approximate experimental conditions, additional simulations were run using generalized born (GB) implicit solvation with single switching and a NaCl concentration of 150 mM [37]. Studies on the flexibility of HP1γ required performance of two simulations, one at 100 ps and another at 2 ns.

### Linear motif analysis for post-translational modifications, protein–protein interaction domains, protein–protein interaction motifs

The presence of a nuclear localization signal (NLS) was derived by combining linear motifs analysis using PsortII, confirming the similarity with other NLSs by virtual peptide display method using Prints [38]. The potential of the IDR1 and IDR2 for binding to DNA was predicted using DP-Bind [39]. Prediction of post-translational modification sites on the CBX isoforms was performed by compiling and statistically scoring linear motifs for phosphorylation, acetylation, methylation, ubiquitination, and sumoylation as predicted by 20 different software programs. The software used to predict phosphorylation were NetPhosK 1.0 [40], NetPhos 2.0 [40], Kinasephos 2.0 [41], DIPHOS [42], PhosphoSVM [43], Scansite, Musite [44], and PPSP [45]. Acetylation sites were predicted using PAIL [46], ASEB [47], BRABSB-PHKA [48], LysAcet [49], and LAceP [50]. Methylation sites were predicted using BPB-PPMS [51] and MASA [52]. Ubiquitination sites were predicted using BDM-PUB [53], CKSAAP UbSite [54], and UbPred [55]. Sumoylation sites were predicted using GPS-SUMO [56] and SUMOplot (http://www.abgent.com/sumoplot/). Results from these predictions were then compiled and statistically scored to assign specificity potential to sites that were predicted to undergo modification in HP1 proteins. Briefly, for each distinct software, we considered sites for which the prediction score was above the cut-off that had been derived using a training set of modified sequences that have been experimentally validated. Subsequently, we developed a meta-prediction score (MPS) by assigning a maximum score of 1 to sites that were predicted by all of the programs cited. Scores for other programs were numerically expressed relative to this maximum score. Results of these predictions were then compared to experimentally validated sites listed in PhosphositePlus [57] and PHOSIDA [58] databases to define whether all predicted sites have also been found in large-scale OMICs analyses.

### Immunoprecipitation of HP1γ complexes and mass spectrometry

Subconfluent HeLa cells were lysed and immunoprecipitation of HP1γ was performed using the Pierce Crosslink Magnetic IP/Co-IP Kit according to the manufacturer’s instructions. HP1γ antibody (Abcam) was cross-linked to the Protein A/G magnetic beads using disuccinimidyl suberate (DSS) to minimize IgG contamination in the final elution. The immunoprecipitated HP1γ complexes were resolved on a 4–15 % Criterion Tris–HCl polyacrylamide gel (Bio-Rad) and stained with Bio-Safe Coomassie Stain (Bio-Rad) according to the manufacturer’s recommendations. Subsequently, bands were selected for excision and processed for nano high-pressure liquid chromatography electrospray tandem mass spectrometry (nano-LC-ESI-MS/MS) by the Mayo Medical Genome Facility Proteomics Core.

### Electron microscopy

For visualizing the shape and contour of the HP1γ dimer, we produced and purified an N-terminal 6×His-tagged recombinant form of this protein using the pET vector system (Novagen, CA). The HP1γ-encoding plasmid was grown in DE3 BL21 bacteria cells overnight and induced with 0.5 mM IPTG for 90 min at 32 °C. The recombinant protein was purified using the Thermo Scientific HisPur Cobalt Resin Kit according to the manufacturer’s instructions. Protein was dialyzed overnight and concentrated to a final concentration of 1 mg/ml. For visualization at the electron microscopy level, 10 μl of the purified protein solution was placed on the surface of glow-discharged formvar carbon-coated grids. After 30 s, the grids were blotted and stained for 30 s in 1 % uranyl acetate. Micrographs were acquired using a JEOL, JEM-1400Plus TEM at 80-kV accelerating voltage, equipped with a Gatan Orius 832 camera.

## Results

### Building a high-resolution molecular model of full-length human HP1γ

We sought to build a model to enhance our understanding of the structure and molecular dynamics of the human full-length HP1γ. The goal of our study was to use Short Linear Motifs (SLiMs) algorithms, homology modeling, threading, in silico mutations, docking, and molecular dynamics simulations to infer biochemical and biophysical information contained particularly within those regions of the protein for which the structure has not been determined. These regions, which together encompass 41.5 % of the protein, correspond to the 31 a.a. N-terminal and 12 a.a. C-terminal tail, as well as the 33 a.a. peptide that links the two known globular domains. Several observations led to modeling these regions of HP1γ as Intrinsically Disordered Regions (IDRs) 1, 2, and 3 (Fig. 1a), a fact that subsequent MD simulations later demonstrated. Initially, hydropathic analyses, shown in Fig. 1b, indicated that these regions display a high polar-to-hydrophobic ratio of residues, a characteristic of Intrinsically Disordered Protein Regions [59]. Furthermore, several order-to-disorder prediction algorithms, such as PrDOS [13], metaPrDOS [14], POODLE [15], DISpro [16], DisEMBL [17], IUPred [18], PONDR-FIT [19], PreDisorder [20], OnD-CRF [21], RONN [22], FoldIndex [23], DISOclust [24], and GlobPlot2 [25], revealed a large propensity for each of the three regions to remain unfolded as an IDR in solution (Fig 1c; Supplementary Fig. 1a–c). As a negative control, we performed the same disorder meta-prediction on a helical region of the HP1γ chromoshadow domain (PQIVIAFYEER; residues 161–171). The results of this meta-prediction show that most of this region is ordered, as opposed to the IDRs (Supplementary Fig. 1e). Homology-based modeling of the HP1γ IDR2 domain, using the *Xenopus laev*is N1N2 phosphoprotein structure as a template (PDB: 1PJN), also indicated its tendency to adopt a random coil conformation (Fig. 1e). We chose N1N2 as a template since it was used in previous structural studies to determine the specificity of α-importin for a variety of nuclear localization signal sequences [32]. The structure of the N-terminal tail (IDR1), also as a random coil, was derived from threading results (Fig. 1d), which were congruent with the predictions of disorder (Fig. 1c; Supplementary Fig. 1a–c). Similar random coil assignments to the structure of the HP1γ linker region (IDR2) and the C-terminal tail (IDR3) were obtained by threading (Fig. 1d–f) and were congruent with predictions of disorder (Supplementary Fig. 1a–c).

**Fig. 2:**
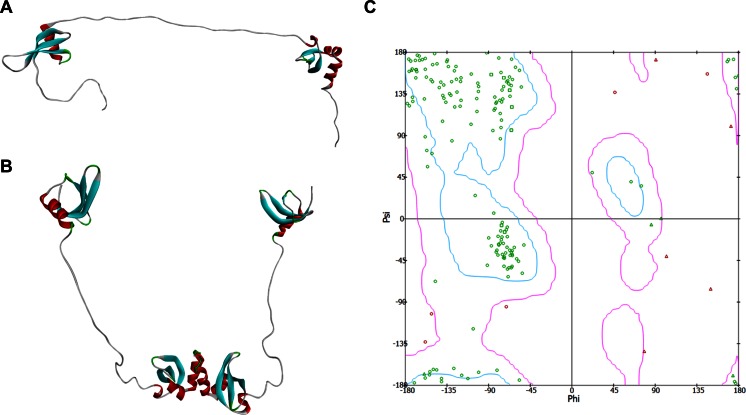
Constructed models of the HP1γ monomer and homodimer. **a** Full-length model of HP1γ was generated by joining the globular domains (CD and CSD) with the IDRs using the Builder function in Discovery Studio Client 4.0. **b** Constructed model of the HP1γ homodimer. **c** Ramachandran plot reveals 97 % of the residues for this model to be in favored or allowed regions

Thus, together, using these collective inferences, we built the final model of the full-length HP1γ monomer by joining all of these fragments using the bonding function of Builder (Fig. 2a) [29]. A model for the HP1γ-HP1γ homodimer was built by docking the monomer using homology-based rules derived from evolutionarily conserved chromoshadow domains (overall identity of 74.6 and 100 % in the residues used for docking; Fig. 2b; PDB: 3DM1). The models were then refined, first by appropriate energy minimization (see Materials and methods) using a harmonic restraint, with a scaled force constant of 10 kcal/mol, on both globular domains. This minimization step was repeated after removal of the harmonic restraint. To estimate the quality of the monomer model, we generated Ramachandran plots (Psi vs. Phi angles plot) using PROCHECK [60], which revealed that 97 % of residues were in favored and allowed regions (Fig. 2c). Taking into consideration that the globular domains are derived from high-resolution structures (PDB: 3KUP and 3DM1) as well as the congruency of many approaches that model the rest of the protein as IDRs, we believe that this model is highly reliable and useful.

### Structural and dynamics properties of the full-length HP1γ molecule

**Fig. 3:**
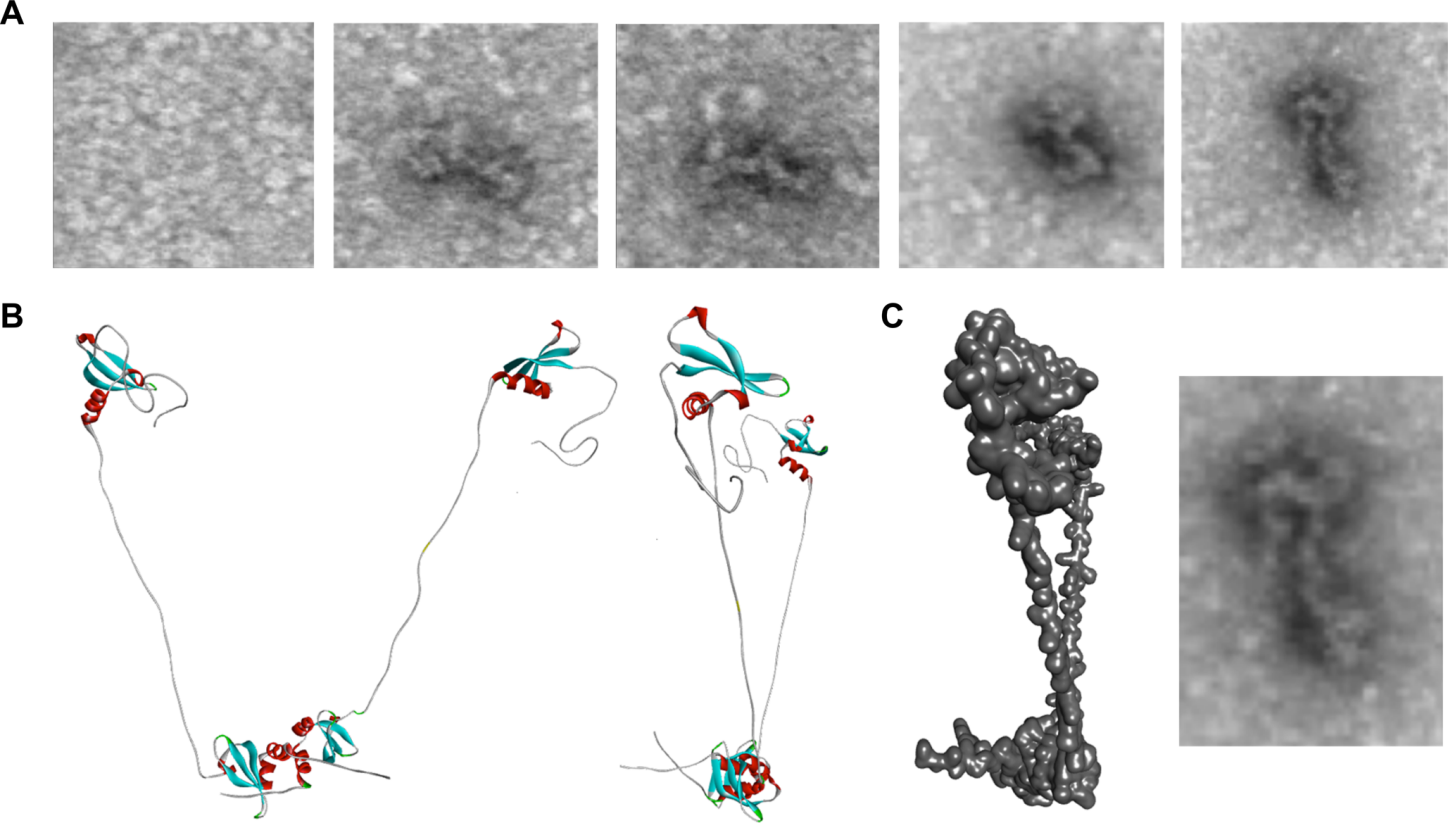
Electron microscopy validates the shape of the HP1γ homodimer. **a** Electron microscopy (EM) images of purified HP1γ. **b** Homology-based model of the HP1γ homodimer. **c** Superimposition of the homodimer structure shows that the predicted model fits nicely with the shape determined by EM imaging. Similar observations have been recently obtained for the yeast HP1 proteins, SWI6 [9]

In order to assess the structural and dynamic properties of our full-length model, we first calculated the length of HP1γ, as modeled in its most extended conformation possible, using the Distance Monitor feature of Discovery Studio [29]. We computed the total comparative length of HP1γ with similar models built for HP1α and HP1β by adding the length of the chromodomain, chromoshadow domain, and IDRs, as well as the Connolly surface, which illustrates the solvent accessibility of the molecules (accessible surface area, ASA). Other comparative properties of these HP1 monomers, including volume, surface area, sphericity, center of mass, solvation energy, and electrostatic potential, were measured using VADAR [30] and the 3 V Volume Calculator [31]. A comparison of these properties is listed in Supplementary Table 1. A detail of the homodimerization interface for the HP1γ homodimer, as observed in our system, is also given in the supplementary material (Supplementary Table 2). Experimentally, we found that a model for the HP1γ homodimer fits nicely with the shape of HP1γ particles imaged using negative staining electron microscopy (Fig. 3a–c), which considerably resembled electron microscopy recently reported for the yeast HP1γ ortholog, SWI6 [9] as well as SAXS experiments on HP1β [61].

**Fig. 4:**
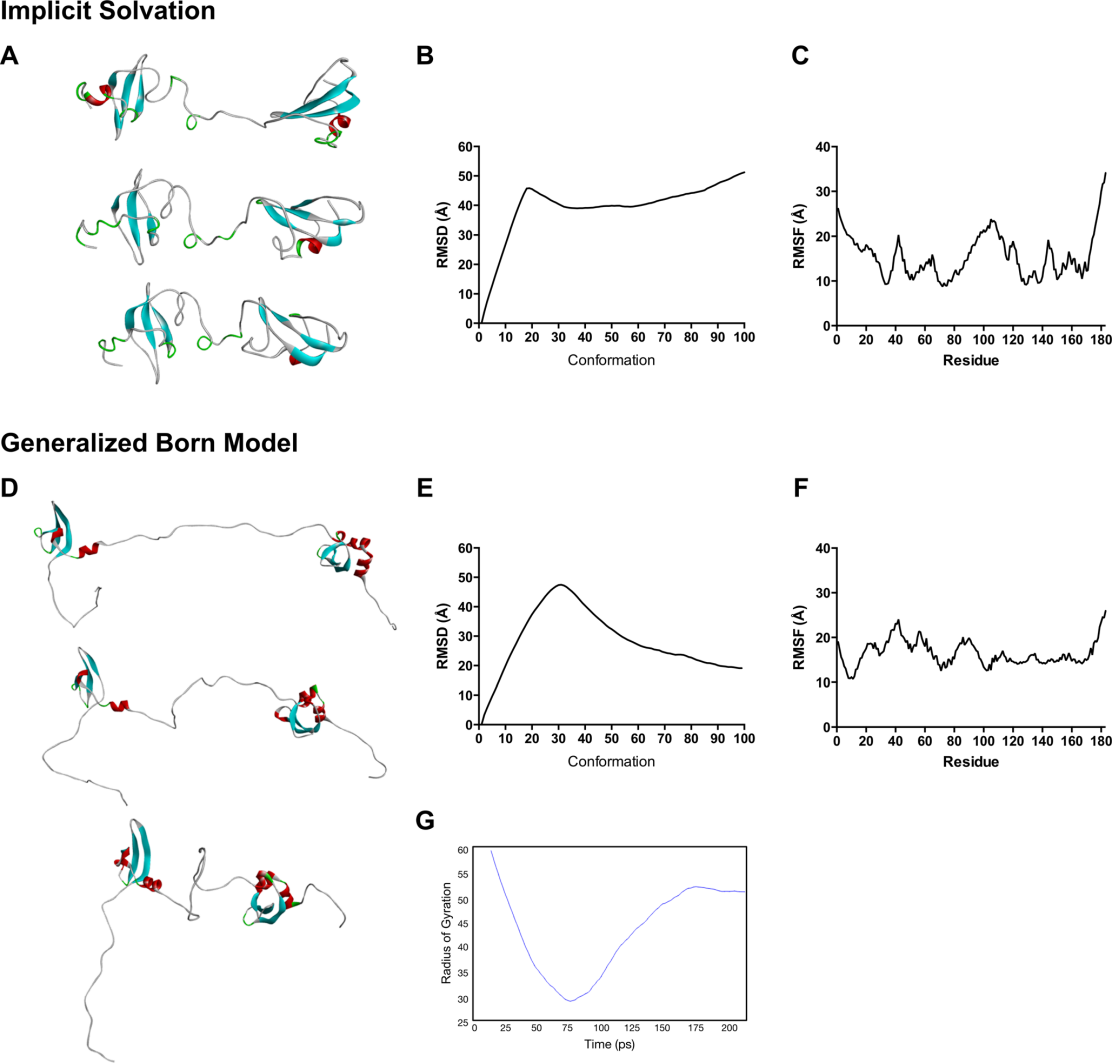
Molecular dynamics simulations of the HP1γ monomer. In order to gain further insight into the biochemical behavior of HP1γ, we subsequently utilized molecular dynamics simulations to perform a conformational search that might reflect the biophysical behavior of HP1 proteins. **a** An assemblage of conformers obtained during a short 100-ps MD simulation. Analysis of the trajectories obtained through this approach reveals that the highly flexible linker has the ability to shorten the length of this protein very rapidly. **b** A numerical representation of the flexibility and mobility of this protein during the simulation time was obtained by calculating the root-mean-square deviation (RMSD) and **c** root-mean-square fluctuation (RMSF). **d** Conformational sampling of the 2-ns GB model simulation highlights the same characteristic flexibility of IDR2 as the implicit solvent model. These results are congruent with the structural bioinformatics analyses, which suggest that the linker region has the highest propensity toward disorder. **e**, **f** RMSD and RMSF values for the generalized born (GB) simulation are also represented. **g** Radius of gyration calculation for the generalized born (GB) simulation

Subsequently, we performed a conformational search that might reflect the biophysical behavior of HP1γ using molecular dynamics simulations. Figure 4a represents an assemblage of conformers obtained during a short MD simulation. These initial simulations were run with implicit solvent models due to the computational expense of explicit solvent models. To address this issue, we ran additional MD simulations using a generalized born (GB) model with single switching to allow for simulation under a better approximation of experimental conditions (150 mM NaCl). After 2 ns of this simulation, the molecule had completely coiled on itself and moved freely with its three IDRs widely sampling the conformation space (Fig. 4b). RMSD and RMSF calculations provided a comparative numerical representation of the flexibility and mobility of the different domains across simulation time. The HP1γ IDRs displayed the highest RMSF values, suggesting that they had the highest degree of flexibility. These numerical results were consistent with the visual data provided by the conformational sampling (Fig. 4c–d) Radius of gyration calculations revealed a repetition of this movement across simulation time (Fig. 4e). Thus, the highly flexible IDR2 endows HP1γ with the ability to shorten its length very rapidly to adopt a final stable conformation where both globular domains come into close proximity with each other (Fig. 4a–b). Our short MD simulations display that HP1γ can sample a wide conformational space by populating an extended ensemble. Longer MD simulations, performed at 2 ns and 10 ns, both reveal that due to the high flexibility of the hinge region, the two globular domains, CD and CSD, come close together with time but do not make direct contact, a fact that would facilitate the spatial search by the two domains for their binding partners (Supplementary Fig. 2). Notably, a similar behavior for HP1β and the yeast HP1 protein SWI6 has been recently observed using experimental techniques. This behavior by both isoforms is likely to be important for HP1 molecules to bridge nearby nucleosomes to form heterochromatin [62–64], and to recruit different binding partners that regulate chromatin functions [2]. Thus, in summary, molecular modeling reveals HP1γ as an elongated molecule, which in spite of having key globular domains, for the most part behaves as an IDP endowed with a high level of dynamic flexibility. This model is congruent with a significant number of biochemical studies and helps to predict additional experimental data, such as our own electron microscopy data (Fig. 3a–c). Therefore, this model is an attractive tool for studying the molecular behavior of HP1γ in silico, using molecular mechanics and dynamics combined with mutational analyses, different solvation environments, and energetic calculations.

### The IDR2 domain of HP1γ mediates protein–protein interactions: heterodimerization with α-importin

**Fig. 5:**
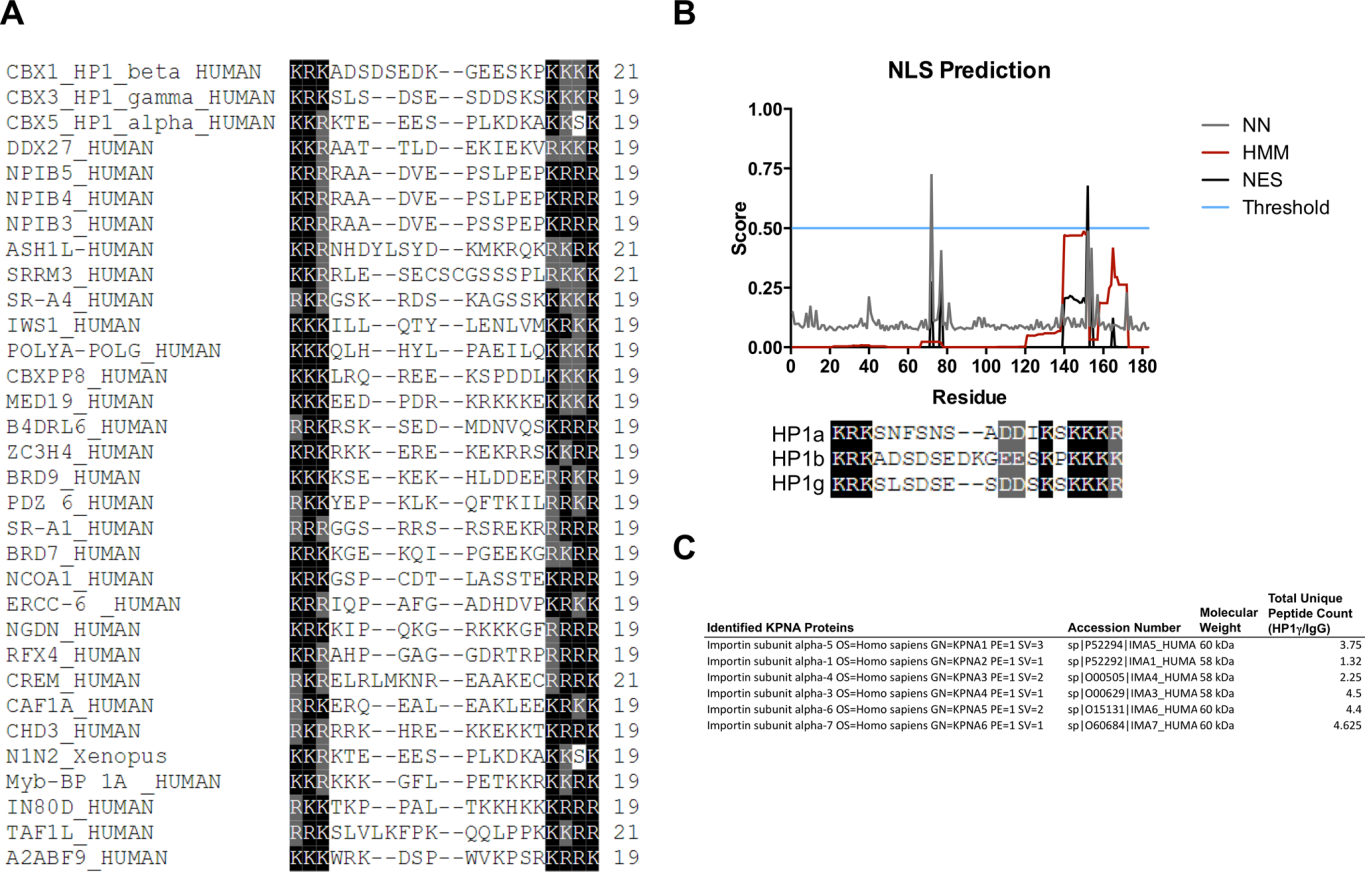
Identification of a nuclear localization signal in HP1γ. **a** Sequence comparison of HP1 NLS motifs with other validated NLS sequences was performed with MUSCLE [76]. This comparison validates the presence of these motifs in these proteins. **b** Since no mutational analyses exist that provide clues as to the cellular and molecular function of this region of the HP1 proteins, we applied the PsortII algorithm to determine that they primarily form a bipartite NLS that conforms to the consensus sequence (K/R)(K/R)*X*10–12(K/R)3/5, where (K/R)3/5 represents at least three of either lysine or arginine of five consecutive amino acids. The fact that the linkers of HP1 proteins are primarily composed of NLS motifs is in agreement with the results of our sequence-to-structural predictions, since all structural studies performed to date for this type of domains reveal their high degree of flexibility and tendency to disorder. **c** Immunoprecipitation of HP1γ followed by mass spectrometry demonstrated that this protein co-purifies in complex with the NLS receptor proteins, α-importins

**Fig. 6:**
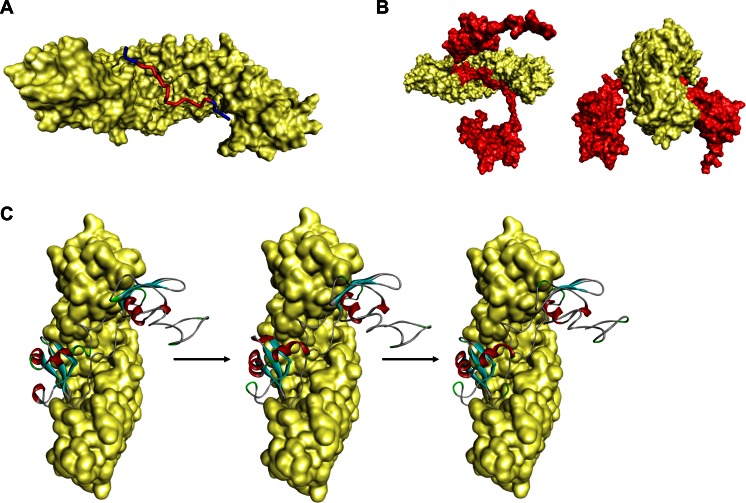
Modeling and simulation of the HP1γ-α-importin complex. **a** Model of IDR docked to the binding site of α-importin. The minor site specifically binds to the N-terminal basic cluster KR (represented in *blue*), and the larger, C-terminal basic cluster KRKK (represented in *blue*) binds to the major site. **b** Model of HP1γ bound to α-importin. The steric environments created by this interaction leaves room for both the chromodomain and chromoshadowdomain of HP1γ to extend outward and downward from the intermolecular interphase. **c** Molecular dynamics simulation of the complex. Unlike the isolated HP1γ simulation, binding to α-importin greatly restricts the disordered motion of IDR2

HP1γ has been previously described to be present in the cytoplasm and translocate to the nucleus where it binds to nucleosomes located at promoters [65] and gene bodies [2]. However, how these proteins are transported to the nucleus has not been defined. Functional SLiM prediction algorithms such as PsortII [38] demonstrated that IDR2 HP1γ region primarily forms a bipartite NLS that conforms to the consensus sequence (K/R)(K/R)*X*_10–12_(K/R)_3/5_, where (K/R)_3/5_ represents at least three of either lysine or arginine in five consecutive amino acids (Fig. 5a–b) [66]. Complementary information was gathered by searching PROSITE [67] with the equivalent X-X-X-X-[KRT]-[KA]-R-K-[ST]-X-X-X-X-syntax-based seed, which matched the nuclear localization signal of many known chromatin proteins (Fig. 5a). Experimentally, these results are consistent with results from our proteomic experiments shown in Fig. 5c, in which immunoprecipitation of HP1γ followed by mass spectrometry demonstrated that this protein co-purifies in complex with the NLS receptor proteins, α-importins. Thus, we used docking, minimization, and molecular dynamic simulations to develop the first model for an HP1γ-α-importin complex, based on the solved structure of the bipartite NLS from nucleophosmin (N1N2). The rules for docking NLS to α−importin have been extensively validated by both experimental and modeling studies [32, 68]. Thus, in this study the IDR2 region was modeled first by homology to the conformation described for the isolated N1N2 NLS (PDB: 1PJN), which is a paradigm for docking homologous peptides to α−importin [32]. For this purpose, we used the crystal structure of an N-terminal truncated mouse α-importin lacking residues 1–69, as these residues are responsible for autoinhibition. In this model, we observe α-importin as a single elongated domain built from ten Arm structural repeats, each containing three α helices (H1, H2, and H3) connected by loops (Fig. 6a). Both the N-terminal and C-terminal basic stretches of amino acids within the HP1γ linker (IDR2) primarily interact with α-importin via salt bridges while the intervening residues additionally contribute to the complex by establishing hydrogen bonds. The two basic clusters of the HP1γ linker bind to two separate well-defined binding sites on the surface of the α-importin molecule, referred to as the minor and major sites (Fig. 6a). The minor site specifically binds to the N-terminal basic cluster KR, and the larger, C-terminal basic cluster KRKK binds to the major site. Notably, HP1γ fits nicely within the binding pocket of α-importin, with a high complementarity in charge and shape. The steric environments created by this binding mode provide space for both the chromodomain and chromoshadow domain to extend outward and downward from the intermolecular interface (Fig. 6b). The details of these interactions are listed in Supplementary Table 3 and are represented graphically in Fig. 6a–b. We subsequently refined this model by molecular dynamics simulations (Fig. 6c). Compared with the model of an isolated HP1γ molecule, binding of this protein to importin restricts its motion. In addition, compared with the first complex of its isolated NLS, binding of the IDR2 of the full-length HP1γ to α-importin is stabilized by additional bonds. Taking into consideration that, as shown below, this region is amenable to extensive posttranslational modifications, which may interfere or enhance these intermolecular interactions, this model will facilitate future mechanistic understanding of how signaling cascades influence the nuclear transport and thus the function of HP1γ and, by homology, how its other isoforms (HP1α and HP1β) as well as its orthologs (e.g., SWI6) are transported.

### The IDR1 and IDR2 domains of HP1γ mediate protein–DNA interactions

**Fig. 7:**
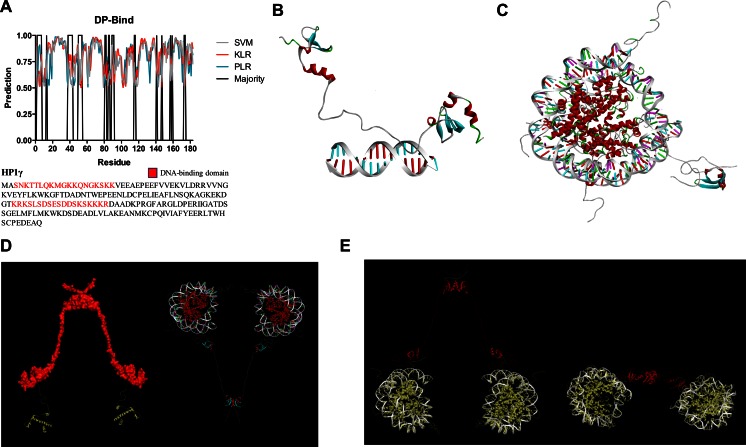
Modeling of HP1γ–DNA complexes. **a** Prediction of DNA-binding residues by DP-Bind. Results of SVM, KLR, and PLR are represented along with the majority or consensus score of the three predictions, showing DNA-binding residues in the three IDR regions. **b** HP1γ–DNA complex generated by DP-Dock. Note how the interaction of IDR2 with B-DNA is consistent with the results of the DP-Bind prediction. **c** Model of IDR1 bound to a single nucleosome. **d** Model of the HP1γ dimer docked to two nucleosomes. **e** MD simulation of the HP1-nucleosome complex shows the PXVXL-domain docked peptide recruited by the HP1γ dimer. The most N-terminal domain of HP1γ contacts the DNA, which is in agreement with experimental data

Next, we used three different yet complementary approaches to identify residues involved in DNA binding by HP1γ [39]. The first method, DP-bind, implements three machine learning methods—support vector machine (SVM), kernel logistic regression (KLR), and penalized logistic regression (PLR)—to predict DNA-binding and RNA-binding residues from primary structure features, including the side-chain p*K*_a_ value, hydrophobicity index, and molecular mass of an amino acid [39]. Figure 7a provides a graphical representation of the results obtained with this approach, which predicted that the two basic clusters (KRKS-(X_9_)-KSKKKR) from the NLS sequence have the potential to interact to DNA. A second basic DNA binding region, present at the most N-terminal region of the protein carrying the sequence SNKTTLQMGKKQNGKSKK was also identified by this method. Interestingly, these predictions are in agreement with experimental data [69], though no details of these interactions currently exist at the atomic resolution level. Subsequently, we sought confirmation of this finding by DP-Dock, another independent unbiased prediction approach, which uses different rules and 3D structural models as input. An added value of DP-Dock is that, if successful, it would allow the generation of structural HP1γ-DNA complexes that can be used for many biochemical and drug-discovery studies. Briefly, DP-Dock uses a nonspecific B-DNA to probe the binding site on a 3D model of a protein that is known to bind DNA but for which the specific amino acid to nucleic acid base contacts are unknown [33]. Given the structure of a DNA-binding protein, the method first automatically generates an ensemble of protein–DNA complexes obtained by rigid-body docking with nonspecific canonical B-DNA molecules with the sequence A10–T10 [33]. Models are subsequently selected by clustering and ranking them according to their DNA–protein interfacial energies [33]. Figure 7b demonstrates successful generation of an HP1γ–DNA complex where the amino acid to base contacts were primarily given by the same linker region sequence identified through DP-bind (Fig. 7a). Analyses of the protein–DNA interface indicated that residues Asp89, Ser90, Lys91, Ser92, Lys93, Phe105, Gly108, Leu140, Met146, and Lys147 interact with DNA. The ionic and hydrogen-bonding interactions that define the protein–DNA binding interface are listed in Supplementary Table 4. Notably, these residues have been experimentally shown to be involved in DNA binding through the combination of EMSA and site-directed mutagenesis [69]. However, the sequence of DNA used for the DP-dock algorithm is too short for determining whether the N-terminal region of HP1γ, as predicted above by DP-BIND (Fig. 7a) and revealed by experimental methods, also interacts with DNA. Consequently, to gain further insight into this phenomenon, we used a third approach based upon carefully examined HP1γ-nucleosome models after MD simulations. Noteworthy, we found that following 2 ns of simulation, the dynamic HP1-nucleosome contact is characterized by the binding of this N-terminal region of the protein with the DNA duplex. Combined these three methods, DP-BIND, DP-DOCK, and MD simulations of HP1γ-nucleosome complexes reveal that HP1γ can interact with DNA in a sequence-independent manner (Fig. 7c–e). Thus, together, these analyses underscore the importance of the intrinsically disordered regions of HP1γ, not only to support the dynamic behavior of the protein but also to carry information for mediating protein–protein and protein–DNA interactions.

### Post-translational modification of the intrinsically disorder regions of HP1γ have the ability to influence intermolecular interactions and histone mimicry

**Fig. 8:**
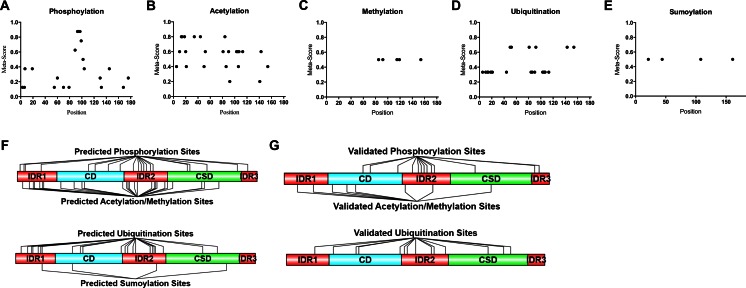
Characterization of IDR2 as the signal integration center through prediction of post-translational modifications. Prediction of post-translational modification sites on HP1γ was performed by compiling and statistically scoring linear motifs for phosphorylation (**a**), acetylation (**b**), methylation (**c**), ubiquitination (**d**), and sumoylation (**e**) as predicted by 20 different software programs. For each program, we considered sites for which the prediction score was above the cut-off that had been derived using a training set of modified sequences that have been experimentally validated. Subsequently, we developed a meta-prediction score by assigning a maximum score of 1 to sites that were predicted by all of the programs cited. Scores for other programs were numerically expressed relative to this maximum score of 1. This analysis revealed that predicted phosphorylation sites have high specificity potential near IDR2 of HP1γ. **f** Graphical representation of predicted post-translational modification sites. Results of this analysis revealed that post-translational modifications have the propensity to occur throughout the entire HP1γ sequence, but appear to be heavily localized to the IDR regions. **g** Graphical representation of experimentally validated post-translational modification sites listed on PhosphositePlus [57] and PHOSIDA [58]

**Fig. 9:**
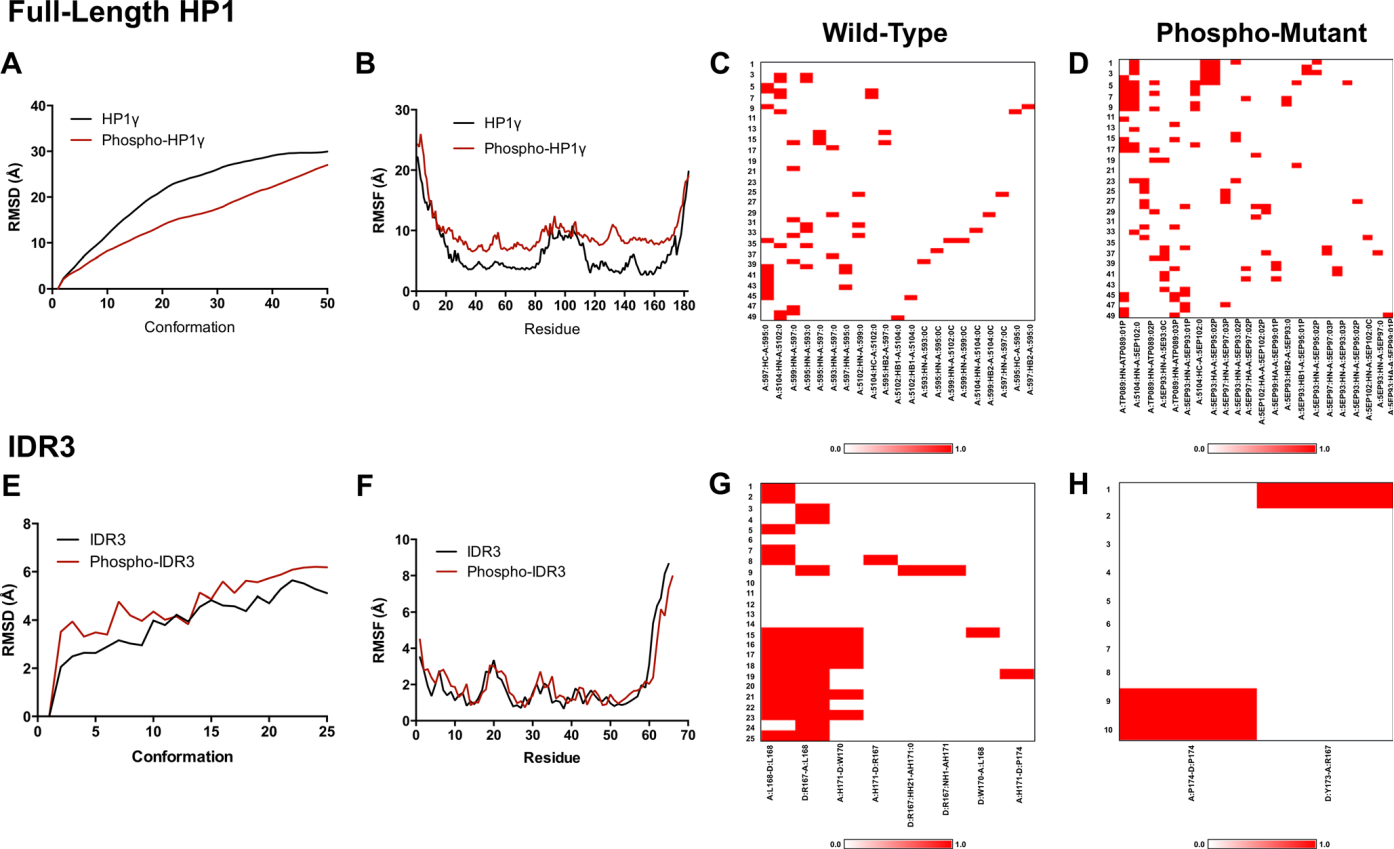
Effects of mutation on the intramolecular binding properties of HP1γ. Molecular dynamics simulations were used to determine the effect of phosphorylation on the stability and intramolecular binding of HP1γ. **a**, **b** Comparative MD simulations of the full-length wild-type HP1γ and phosphorylated HP1γ with the following amino acid positions mutated: 55, 60, 79, 89, 93, 95, 97, 99, 102, and 176. **c**, **d** The simulation results suggest that phosphorylation increases the time-dependent intramolecular binding of the phosphorylated mutant when compared to the wild type. **e**, **f** Comparative MD simulations of the full-length wild-type IDR3 and phosphorylated IDR3 with the following amino acid positions mutated: 169 and 172. **g**, **h** Similar to the full-length HP1γ, these simulation results suggest that phosphorylation increases the time-dependent intramolecular binding of the phosphorylated mutant when compared to the wild type. Together, these results support the use of linear motif analysis to predict post-translational modifications as these simulations suggest a relevant biophysical effect of phosphorylation on the behavior and intramolecular binding of HP1γ

Since HP1 isoforms function in the regulation of cancer-associated gene expression networks, it is important to gain insight into the mechanisms by which these proteins are either activated or inactivated. A number of histone code-like post-translational modifications have been described and validated experimentally [57, 58, 70]. To determine other potential post-translational modification sites that have not been determined, we performed extensive linear motif analyses on HP1γ, using the primary sequence as input in order to gain further insight into the differential regulation of this protein. First, post-translational modifications, such as phosphorylation, acetylation, methylation, ubiquitination, and sumoylation, were predicted using various modification prediction algorithms that create neural networks of potential sites based off a set of experimentally validated sites, support vector machines (SVM), and machine learning methods, such as kernel logistic regression (KLR) and Bayesian decision theory. These sites were then compared to experimentally validated sites listed in PhosphositePlus [57] and PHOSIDA [58] databases. The raw output of these predictions is included in Supplementary Table 5a–e. The results of these analyses are represented graphically in Fig. 8 and reveal that phosphorylation (a), acetylation (b), methylation (c), ubiquitination (d), and sumoylation (e) potentially occur throughout the entire sequence of HP1γ. Furthermore, the analyses predicted several potential post-translational modification sites in IDR2 of HP1γ that have not been validated by mass spectrometry, such as phosphorylation sites at Ser70, Ser79, Thr89, and Ser99. Additionally, acetylation sites were predicted at Lys81, Lys84, Lys103, Lys105, Lys107, Lys113; methylation sites were predicted at Lys85, Arg115, Arg119; ubiquitination sites were predicted at Lys81, Lys84, Lys103, Lys105, Lys107, Lys113; sumoylation sites were predicted at Arg108 (Fig. 8f). While these sites have not been experimentally validated, they provide additional insight into the differential regulation of HP1γ. Since the proteomics experiments were performed on one cell type under one condition, it remains likely that some modification sites from the predicted group integrate signals under conditions that are very different from the culture studies. Thus, both comparisons are justified in this analysis (Fig. 8g). Collectively, the linear motifs present on the linker for nuclear localization, DNA binding, and post-translational modifications, as well as its predicted propensity toward disorder support the hypothesis for the role of IDR2 as the signal integration microdomain for HP1γ. In fact, the predicted sites from this analysis prompted us to examine the effects of post-translational modifications and mutations on the biophysical properties of HP1γ and its interactions with some binding partners. For this purpose, we initially constructed a model of a phosphorylated full-length HP1γ as well as its IDR2 and IDR3. We subsequently ran comparative MD simulations of these models in their phosphorylated and unphosphorylated states (Fig. 9a–b). Details of the mutated amino acid positions are provided in the figure legends (Fig. 9). Results of these simulations are consistent with the observation that phosphorylation of IDR2 restricts HP1γ movement by increasing the time-dependent intramolecular binding (Fig. 9c–d). A similar phenomenon is seen with IDR3, where phosphorylation restricts its movement when compared to the wild-type peptide (Fig. 9e–h). Combined, these results support the relevance of the linear motif analysis by highlighting the biophysical effects of post-translational modifications on the behavior and function of HP1γ.

**Fig. 10:**
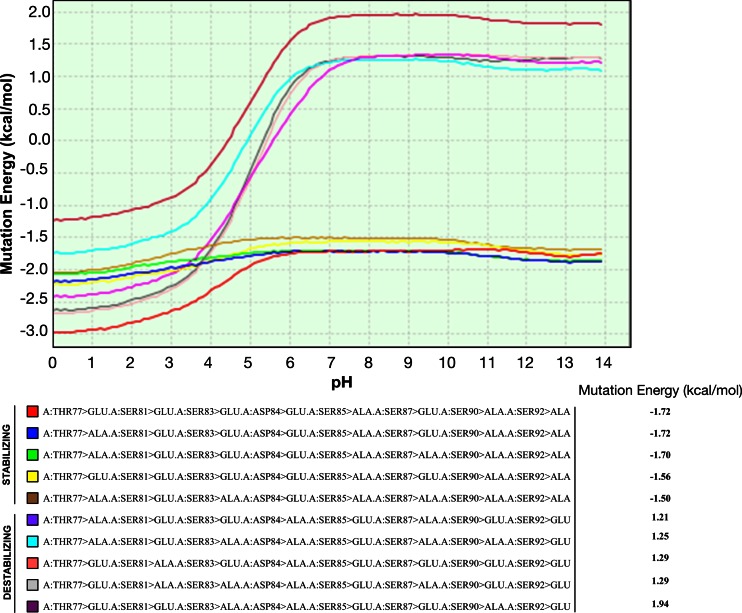
Combinatorial amino acid scanning mutagenesis reveals effect of phosphorylation on the binding affinity between HP1γ and α-importin. Residues in IDR2 were changed to either glutamic acid (phospho-mimicking) or alanine (non-phosphorylatable). Calculated mutation energies that were less than −0.5 kcal/mol were considered stabilizing while energies greater than 0.5 kcal/mol were considered destabilizing. Phospho-mimicking mutations displayed lower mutation energy profiles and thus a lower binding affinity for α-importin. Results of this analysis revealed that phosphorylation-mimicking mutations decrease affinity of HP1 for α-importin

In addition, we also analyzed the effect of mutations on the binding affinity between HP1γ and α-importin, by performing combinatorial amino acid scanning mutagenesis on residues in IDR2 using MD simulations. The effect of each mutation on the binding energy was calculated by the following equation DDGbind = DG (mutant) - DG (wild type), where DG is the difference between the free energy of the complex and the free energy of the unbound state. Total free energy was calculated using a GB implicit solvent model and is the weighted sum of the van der Waals and electrostatic interactions as described by Spassov and Yan [71]. Briefly, residues in IDR2 were changed to either glutamic acid or alanine: glutamic acid substitutions served as phosphorylation-mimicking mutations, while alanine substitutions mimicked non-phosphorylated residues. Calculated mutation energies that were less than –0.5 kcal/mol were considered stabilizing while energies greater than 0.5 kcal/mol were considered destabilizing (Fig. 10). Results of this analysis revealed that phosphorylation-mimicking mutations decreased the affinity of HP1γ for α-importin (Fig. 10). Thus, combined, these results demonstrate that the dynamic behavior as well as intermolecular interaction properties of HP1γ can be influenced by post-translational modifications, which, because of its solvent accessibility, occur most frequently in the intrinsically disordered regions of this protein.

**Fig. 11:**
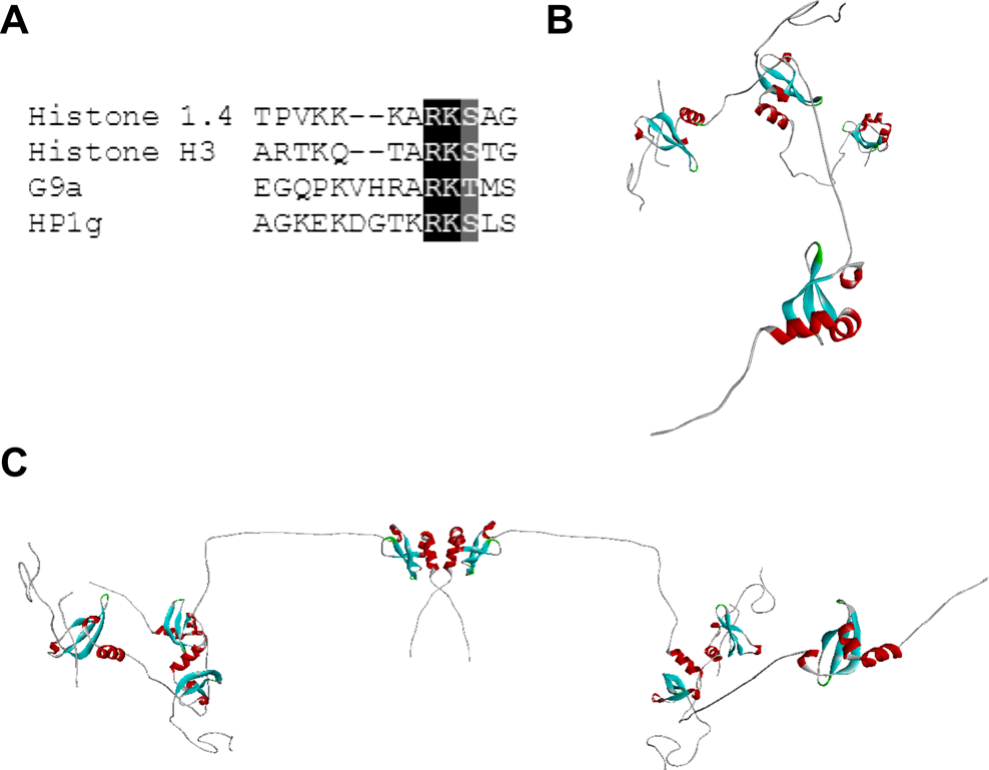
Modeling of the HP1γ auto-inhibited state. **a** Sequence alignment of the histone mimetic peptide of HP1γ (K82) with Histone 3 (K9), G9a (K185), and Histone 1.4 (K26). **b** Homology-based model of HP1γ in its autoinhibited state. **c** Homology-based model of the autoinhibited HP1γ homodimer. Together, these highlight the hypothesis that the ability of HP1γ to function as a histone mark reader is inhibited when both of its chromodomains are used to bind to additional monomers

Lastly, we sought to gain further insight at the atomic level into an observation that has been made by our laboratory and others, which revealed that HP1γ provides a useful example of histone mimicry. Histone mimicry refers to the fact that small linear motifs in non-histone proteins, when appropriately modified, can mimic histone marks and be recognized by histone code writers, readers, and erasers. The structural basis of this phenomenon has also been elucidated for Histone 3 (K9), G9a (K185), and Histone 1.4 (K26) [9, 72]. Though the presence of a histone mimetic peptide within HP1γ (K82) has also been shown biochemically [73], the rules and functional consequences of its interaction with chromodomains remain to be characterized (Fig. 11a). Toward this end, we used a homology-based approach to model the interaction of the HP1γ monomer to its own histone mimicking peptide (Fig. 11b). We modeled the effects that K82 methylation can have on regulating chromodomain–IDR2 interactions. In doing so, we realized that the ability of the HP1γ homodimer to function as a histone mark reader would be inhibited when both of its chromodomains are used to bind to additional monomers, in isolation or as part of another dimer (Fig. 11c). This observation is in agreement with previous biochemical data showing oligomerization of HP1 molecules from yeast,* Drosophila*, and mammals [9, 62]. More importantly, the ability of HP1γ to autoinhibit through histone mimicry is similar to the behavior of *S. pombe* HP1 (SWI6), recently derived from elegant kinetic studies [9]. SWI6 also contains a histone-mimicking peptide, though different in sequence and location from that of HP1γ. Since the sequence that mediates this event [9] is present in HP1 molecules from many species, this model may help to explain an evolutionarily conserved mechanism for the regulation of this type of histone reader.

In summary, at the onset of the current study, most of the structural considerations related to HP1γ had been confined to its globular chromo and chromoshadow domains. However, little was known about how the rest of the primary sequence influenced the behavior of HP1γ. Using several methodologies germane to structural bioinformatics, modeling, docking, dynamics, and mutational analyses, we have gathered evidence that supports a critical contribution of intrinsically disorder regions to define the connectivity, dynamic flexibility, and intermolecular interactions of this protein. This new knowledge, therefore, significantly contributes to further our understanding of the biophysical properties and biochemical behavior of this important epigenetic regulator.

## Discussion

The current work was initiated as a means to extend our understanding of the molecular properties of HP1γ. However, based on homology and evolutionary conservation of this protein to other members of its family, our models are likely to be applicable to isoforms and orthologs of HP1γ. HP1 proteins are among the most widely characterized epigenetic regulators with many of their functions being conserved throughout evolution [2]. HP1γ associates with the development of human diseases, including many forms of deadly cancers [3, 4]. Recent studies have applied state-of-the-art biophysical methods to solve the structure of this protein as to advance our understanding of the basic biochemical mechanisms underlying its function and the hope that these efforts will aid in the future design of small drug inhibitors. These studies produced the structure of both the chromodomain and chromoshadow domain [11, 74]. In spite of this useful information, no reliable full-length model for HP1γ yet exists. Notably, however, extensive biochemical studies indicate that other parts of the protein, namely the most N- and C-terminal regions as well as the linker, which joins the chromo and chromoshadow domains, may contribute to its function. Toward this end, the current study provides information on the molecular behavior of HP1γ that did not exist before by building and characterizing a structural model for this protein. In fact, our study underscores the critical role of the HP1γ IDRs in molecular connectivity, flexibility, protein–protein, and protein–DNA interactions as well as post-translational modifications, which include histone mimicry. Though useful, HP1α and HP1β models were also built but not studied in a dynamic fashion, so as to maintain our focus on HP1γ. We show models for the HP1γ homodimer (Fig. 2b) and the molecule bound to DNA (Fig 7b–c). We also provide an atomic resolution view of the α-importin-HP1γ complex (Fig. 6). We perform, for the first time, an MD simulation of the full-length HP1γ monomer (Fig. 4), in complex with α-importin (Fig. 6c), and with nucleosomes (Fig. 7e). These studies indicate that the intrinsically disordered parts of the protein make the human HP1γ protein highly dynamic, a characteristic that had never been previously defined for this protein. Dynamic flexibility given by this region may allow other domains, such as the chromodomain, to more easily sample the tridimensional space in search for binding partners. Thus, we are optimistic that future studies using experimental techniques may test the validity of this interpretation. This dynamic behavior, however, appears to be restricted when HP1γ forms complexes. MD simulations using harmonically restrained nucleosome particles bound by a single HP1γ dimer show that due to its flexibility, it has the potential to recoil onto the nucleosomes. This activity allows for the recruitment of the HP1-binding domain of SUV39H1 through its contact with nucleosomes. Combined, the building and analyses of these structural models provide a more complete description of the biochemical function of HP1 proteins, as elongated molecules with their two globular domains joined by a flexible linker, which endows them with dynamic flexibility and intermolecular recognition properties. Thus, it becomes important to discuss the accuracy, novelty, and mechanistic contribution of this new information to understanding the biochemical properties of these important epigenetic regulators. Several observations are in agreement with and extend, at a predicted atomic resolution, experimentally derived data, increasing the reliability of the models: (1) HP1γ has the ability to form an NLS-importin complex, which renders it competent to translocate into the nucleus (Fig. 5). (2) Once in the nucleus, HP1γ binds to 3Me-K9H3 and nucleosomal DNA (Fig. 7). (3) The protein is heavily marked by post-translational modifications, some of them playing a significant role in the regulation of histone mimicry. (4) Similar to its yeast homolog, the histone mimetic peptide within the linker region of HP1γ can be recognized by the chromodomain of this protein, a phenomenon which should inhibit its binding to histone marks. (5) The model suggests that the largest number of post-translational modifications map to the intrinsically disorder regions of the protein, which are more surface exposed. Thus, to our knowledge, when combined, these considerations make the current study novel and important.

Modeling of disordered proteins, such as HP1γ, is challenging as their structure cannot be represented by a single, derived conformation. These highly flexible molecules sample a multitude of conformations; both expanded and collapsed in nature. Thus, several restrictions were applied in the generation of our model. First, the structure of both the model for the monomer and dimer presented here for HP1γ is in agreement with homology-based data available from structural NMR and SAXS data recently made available for HP1β [61]. This model for the HP1γ monomer complexed nicely with α-importin via the IDR2 region, allowing the N-terminal and C-terminal globular domain to protrude out of the complex without steric hindrances (Fig. 6b). Congruently, the model of the dimer was built by docking the chromodomain of individual monomers, rather than stitching domains from docked chromodomains. This method leads the N-terminal IDRs and globular domain to adopt a “lobster claw” configuration, which is in agreement with structural data for the highly homologous protein HP1β [75] and yeast SWI6 [9]. It is also true that a single conformation cannot be considered for either the monomer or the dimer. For this reason, we performed conformational sampling using molecular dynamic simulations. Thus, our data is in agreement with the structure of homologous monomers and dimers from human homologues and yeast orthologues, along with their numerous conformations carefully derived from MD simulations, to faithfully represent the structure expected for HP1γ. Further modeling studies using longer MD simulations and coarse-grained models may lend more insight into the biochemical behavior or HP1γ and its complexes.

In conclusion, force field-supported, molecular mechanic calculations and analyses of molecular dynamics simulations infer that a significant amount of structure-to-function information is contained within the less studied regions of HP1γ. The intrinsically disordered properties of these regions endow the entire molecule with a highly dynamic behavior, intermolecular recognition properties, and the ability to receive signals from several intracellular signaling cascades. Since HP1γ plays a key role in normal epigenetics and cancers, the data and models here reported have current and future applications for better understanding biological and pathobiological functions of this protein. By analogy, this data on HP1γ may also inspire both experimental and in silico testable hypotheses regarding the function of the closest members of this family of proteins.

## Electronic supplementary material

Supplementary Fig 1 Disorder meta-prediction for IDR1, IDR2, and IDR3. Multiple sequence-based disorder prediction algorithms were used to predict the propensity of the linker region toward disorder in solution. Disorder probability values above the cut-off value of 0.5 are considered to be disordered. **d** Additional disorder predictions for IDR2 were performed using RONN, FoldIndex, DISOclust, and GlobPlot2. **e** Additionally, the same disorder meta-prediction was used on a coiled region of the HP1γ chromoshadow domain (PQIVIAFYEER; residue 161-171) as a negative control. The results of this meta-prediction show that most of this region is ordered as opposed to the IDRs. (GIF 83 kb)

High Resolution (TIFF 2772 kb)

Supplementary Fig 2 Extended molecular dynamics simulations of the HP1γ monomer. **a**, **b** A 10-ns MD simulation was performed on the HP1 monomer with implicit-solvation. The temperature and total energy profiles are represented. **c** A numerical representation of the flexibility and mobility of this protein during the simulation time was obtained by calculating the root-mean-square deviation (RMSD) and **d** root-mean-square fluctuation (RMSF). **e** Radius of gyration calculation for the generalized born (GB) simulation. **f** Assemblage of conformers obtained during a short 10-ns MD simulation. (GIF 111 kb)

High Resolution (TIFF 4010 kb)

Supplementary Table 1 Calculated biophysical properties of HP1 monomers. Properties of the HP1 monomers in their most extended conformations were calculated using Discovery Studio, VADAR, and the 3 V Volume Calculator [29–31]. HP1γ measured 18.3 nm, comparable to similar models of its closest homologues, HP1β (17.8 nm), and HP1α (21.0 nm) (Supplementary Table 1). The calculations on these models suggest that the isoforms are similar in mass, volume, length, sphericity, and effective radius. However, they differ in their electrostatic potential and solvation energies. (XLSX 9 kb)

Supplementary Table 2 Bonding patterns of the HP1γ-dimer. Results of interface analysis performed on the HP1γ- HP1γ complex in order to investigate contact residues between the monomers. The complex was subjected to a 2,000-step minimization using steepest descent followed by a 2,000-step conjugated gradient minimization. Contact residues between HP1γ monomers were analyzed by defining an interface as a contact area with a maximum salt-bridge distance of 5.0 Å. (XLSX 10 kb)

Supplementary Table 3 Bonding patterns of the HP1γ-α-importin complex. Results of interface analysis performed on the wild-type HP1γ– α-importin complex in order to investigate contact residues between HP1γ and α-importin. The complex was subjected to a 2,000-step minimization using steepest descent followed by a 2,000-step conjugated gradient minimization. Contact residues between HP1γ and α-importin were analyzed by defining an interface as a contact area with a maximum salt-bridge distance of 5.0 Å. (XLSX 14 kb)

Supplementary Table 4 Bonding patterns of the HP1γ–DNA complex. Results of interface analysis performed on the wild-type HP1γ–DNA complex in order to investigate contact residues between HP1γ and DNA. The complex was subjected to a 2,000-step minimization using steepest descent followed by a 2,000-step conjugated gradient minimization with harmonic restraints on all nucleic acid groups. Contact residues between HP1γ and DNA were analyzed by defining an interface as a contact area with a maximum salt-bridge distance of 5.0 Å. (XLSX 12 kb)

Supplementary Table 5 Linear motif analysis data. Raw outputs for the individual programs used in the linear motif analysis. Details of the meta-score calculation can be found in the supplementary text. (XLSX 17 kb)

Supplemental Text 1 (DOC 22 kb)

## References

[1] Eissenberg JC, James T, Foster-Hartnett DM, Hartnett T, Ngan V, Elgin SC (1990). Mutation in a heterochromatin-specific chromosomal protein is associated with suppression of position-effect variegation in* Drosophila melanogaster*. Proc Natl Acad Sci U S A.

[2] Lomberk G, Wallrath L, Urrutia R (2006). The Heterochromatin Protein 1 family. Genome Biol.

[3] Velez G, Urrutia R, Lomberk G (2013). Critical role of the HP1-histone methyl transferase pathways in cancer epigenetics. Med Epigenet.

[4] Dialynas GK, Vitalini M, Wallrath LL (2008). Linking Heterochromatin Protein 1 (HP1) to cancer progression. Mutat Res.

[5] Bannister AJ, Zegerman P, Partridge JF, Miska EA, Thomas JO, Allshire RC, Kouzarides T (2001). Selective recognition of methylated lysine 9 on histone H3 by the HP1 chromo domain. Nature.

[6] Lachner M, O’Carroll D, Rea S, Mechtler K, Jenuwein T (2001). Methylation of histone H3 lysine 9 creates a binding site for HP1 proteins. Nature.

[7] Daujat S (2005). HP1 binds specifically to Lys26-methylated histone H1.4, whereas simultaneous Ser27 phosphorylation blocks HP1 binding. J Biol Chem.

[8] Aasland R, Stewart A (2005). The chromo shadow domain, a second chromo domain in heterochromatin-binding protein 1, HP1. Nucleic Acids Res.

[9] Canzio D (2013). A conformational switch in HP1 releases auto-inhibition to drive heterochromatin assembly. Nature.

[10] Lomberk G (2012). Sequence-specific recruitment of heterochromatin protein 1 via interaction with Krüppel-like factor 11, a human transcription factor involved in tumor suppression and metabolic diseases. J Biol Chem.

[11] Brasher S (2000). The structure of mouse HP1 suggests a unique mode of single peptide recognition by the shadow chromo domain dimer. EMBO J.

[12] Thiru A (2004). Structural basis of HP1/PXVXL motif peptide interactions and HP1 localisation to heterochromatin. EMBO J.

[13] Ishida T, Kinoshita K (2007) PrDOS: prediction of disordered protein regions from amino acid sequence. Nucl Acids Res 35(Web Server Issue):W460–W464 10.1093/nar/gkm363 PMC1933209 17567614

[14] Ishida T, Kinoshita K (2008). Prediction of disordered regions in proteins based on the meta approach. Bioinformatics.

[15] Hirose S (2007). POODLE-L: a two-level SVM prediction system for reliably predicting long disordered regions. Bioinformatics.

[16] Cheng J, Sweredoski M, Baldi P (2005). Accurate prediction of protein disordered regions by mining protein structure data. Data Min Knowl Disc.

[17] Linding R (2003). Protein disorder prediction: implications for structural proteomics. Structure.

[18] Dosztanyi Z (2005). IUPred: web server for the prediction of intrinsically unstructured regions of proteins based on estimated energy content. Bioinformatics.

[19] Xue B (2010). PONDR-FIT: a meta-predictor of intrinsically disordered amino acids. Biochim Biophys Acta.

[20] Deng X, Eickholt J, Cheng J (2009). PreDisorder: ab initio sequence-based prediction of protein disordered regions. BMC Bioinf.

[21] Wang L, Sauer U (2008). OnD-CRF: predicting order and disorder in proteins using [corrected] conditional random fields. Bioinformatics.

[22] Yang Z (2005). RONN: the bio-basis function neural network technique applied to the detection of natively disordered regions in proteins. Bioinformatics.

[23] Prilusky J (2005). FoldIndex: a simple tool to predict whether a given protein sequence is intrinsically unfolded. Bioinformatics.

[24] McGuffin L (2008). Intrinsic disorder prediction from the analysis of multiple protein fold recognition models. Bioinformatics.

[25] Linding R (2003). GlobPlot: exploring protein sequences for globularity and disorder. Nucleic Acids Res.

[26] Wu S, Zhang Y (2007). LOMETS: a local meta-threading-server for protein structure prediction. Nucleic Acids Res.

[27] Wu S, Zhang Y (2008). MUSTER: improving protein sequence profile–profile alignments by using multiple sources of structure information. Proteins.

[28] Sali A, Blundell T (1993). Comparative protein modelling by satisfaction of spatial restraints. J Mol Biol.

[29] Inc AS (2012) Discovery studio modeling environment, release 3.5, in Accelrys Discovery Studio. Accelrys Software Inc, San Diego

[30] Willard L (2003). VADAR: a web server for quantitative evaluation of protein structure quality. Nucleic Acids Res.

[31] Voss N, Gerstein M (2010) 3V: cavity, channel and cleft volume calculator and extractor. Nucleic Acids Res 38(Web Server Issue):W555–W562 10.1093/nar/gkq395 PMC2896178 20478824

[32] Fontes M (2003). Structural basis for the specificity of bipartite nuclear localization sequence binding by importin-alpha. J Biol Chem.

[33] Gao M, Skolnick J (2009). From nonspecific DNA–protein encounter complexes to the prediction of DNA–protein interactions. PLoS Comput Biol.

[34] Urrutia R (2014). Evidence supporting the existence of a NUPR1-like family of helix-loop-helix chromatin proteins related to, yet distinct from, AT hook-containing HMG proteins. J Mol Model.

[35] Cornell WD, Cieplak P, Bayly CI, Gould IR, Merz KM, Ferguson DM, Spellmeyer DC, Fox T, Caldwell JW, Kollman PA (1995). A second-generation force field for the simulation of proteins, nucleic acids, and organic molecules. J Am Chem Soc.

[36] Ryckaert JP, Ciccotti G, Berendsen HJC (1977). Numerical integration of the Cartesian equations of motion of a system with constraints: molecular dynamics of* n*-alkanes. J Comput Phys.

[37] Im W, Lee M, Brooks C (2003). Generalized born model with a simple smoothing function. J Comput Chem.

[38] Nakai K, Horton P (1999). PSORT: a program for detecting sorting signals in proteins and predicting their subcellular localization. Trends Biochem Sci.

[39] Hwang S, Gou Z, Kuznetsov IB (2007). DP-Bind: a web server for sequence-based prediction of DNA-binding residues in DNA-binding proteins. Bioinformatics.

[40] Blom N, Sicheritz-Ponten T, Gupta R, Gammeltoft S, Brunak S (2004). Prediction of post-translational glycosylation and phosphorylation of proteins from the amino acid sequence. Proteomics.

[41] Wong YH, Lee T, Liang HK, Huang CM, Wang TY, Yang YH, Chu CH, Huang HD, Ko MT, Hwang JK (2007). KinasePhos 2.0: a web server for identifying protein kinase-specific phosphorylation sites based on sequences and coupling patterns. Nucleic Acids Res.

[42] Iakoucheva LM, Radivojac P, Brown CJ, O’Connor TR, Sikes JG, Obradovic Z, Dunker AK (2004). The importance of intrinsic disorder for protein phosphorylation. Nucleic Acids Res.

[43] Dou Y, Yao B, Zhang C (2014) PhosphoSVM: prediction of phosphorylation sites by integrating various protein sequence attributes with a support vector machine. Amino Acids 46(6):1459–1469 10.1007/s00726-014-1711-5 24623121

[44] Gao J, Thelen J, Dunker AK, Xu D (2010). Musite, a tool for global prediction of general and kinase-specific phosphorylation sites. Mol Cell Proteomics.

[45] Xue Y, Li A, Wang L, Feng H, Yao X (2006). PPSP: prediction of PK-specific phosphorylation site with Bayesian decision theory. BMC Bioinf.

[46] Li A, Xue Y, Jin C, Wang M, Yao X (2006) Prediction of Nε-acetylation on internal lysines implemented in Bayesian Discriminant Method. Biochem Biophys Res Commun 350(4):818–824 10.1016/j.bbrc.2006.08.199 PMC2093955 17045240

[47] Wang L, Du Y, Lu M, Li T (2012). ASEB: a web server for KAT-specific acetylation site prediction. Nucleic Acids Res.

[48] Shao J, Xu D, Hu L, Kwan YW, Wang Y, Kong X, Ngai SM (2012). Systematic analysis of human lysine acetylation proteins and accurate prediction of human lysine acetylation through bi-relative adapted binomial score Bayes feature representation. Mol BioSyst.

[49] Li S, Li H, Li M, Shyr Y, Xie L, Li Y (2009). Improved prediction of lysine acetylation by support vector machines. Protein Pept Lett.

[50] Hou T (2014). LAceP: lysine acetylation site prediction using logistic regression classifiers. PLoS ONE.

[51] Shao J, Xu D, Tsai SN, Wang Y, Ngai SM (2009). Computational identification of protein methylation sites through bi-profile Bayes feature extraction. PLoS ONE.

[52] Shein D (2009). Incorporating structural characteristics for identification of protein methylation sites. J Comput Chem.

[53] Li A, Gao X, Ren J, Jin C, Xue Y (2009) BDM-PUB: computational prediction of protein ubiquitination sites with a Bayesian discriminant method

[54] Chen Z, Chen Y, Wang X, Wang C, Yan R, Zhang Z (2011). Prediction of protein ubiquitination sites by using the composition of k-spaced amino acid pairs. PLoS ONE.

[55] Radivojac P, Vacic V, Haynes C, Cocklin RR, Mohan A, Heyen JW, Goebl MG, Iakoucheva LM (2010). Identification, analysis and prediction of protein ubiquitination sites. Proteins.

[56] Zhao Q, Xie Y, Zheng Y, Jiang S, Liu W, Mu W, Zhao Y, Xue Y, Ren J (2014). GPS-SUMO: a tool for the prediction of sumoylation sites and SUMO-interaction motifs. Nucleic Acids Res.

[57] Hornbeck PV, Kornhauser J, Tkachev S, Zhang B, Skrzypek E, Murray B, Latham V, Sullivan M (2012) PhosphoSitePlus: a comprehensive resource for investigating the structure and function of experimentally determined post-translational modifications in man and mouse. Nucleic Acids Res 40(Database issue):D261–D270 10.1093/nar/gkr1122 PMC3245126 22135298

[58] Gnad F, Gunawardena J, Mann M (2011) PHOSIDA 2011: the posttranslational modification database. Nucleic Acids Res 39(Database Issue):D253–D260 10.1093/nar/gkq1159 PMC3013726 21081558

[59] Wong E, Na D, Gsponer J (2013). On the importance of polar interactions for complexes containing intrinsically disordered proteins. PLoS Comput Biol.

[60] Laskowski RA, MacArthur MW, Moss DS, Thornton JM (1993). PROCHECK - a program to check the stereochemical quality of protein structures. J Appl Crystallogr.

[61] Munari F (2014). Characterization of the effects of phosphorylation by CK2 on the structure and binding properties of human HP1β. FEBS Lett.

[62] Canzio D (2011). Chromodomain-mediated oligomerization of HP1 suggests a nucleosome-bridging mechanism for heterochromatin assembly. Mol Cell.

[63] Munari F (2012). Methylation of lysine 9 in histone H3 directs alternative modes of highly dynamic interaction of heterochromatin protein hHP1β with the nucleosome. J Biol Chem.

[64] Ruthenburg A (2007). Multivalent engagement of chromatin modifications by linked binding modules. Nat Rev Mol Cell Biol.

[65] Smothers J, Henikoff S (2001). The hinge and chromo shadow domain impart distinct targeting of HP1-like proteins. Mol Cell Biol.

[66] Kosugi S (2009). Six classes of nuclear localization signals specific to different binding grooves of importin α. J Biol Chem.

[67] Sigrist CJ, Cerutti L, de Castro E, Langendijk-Genevaux PS, Bulliard V, Bairoch A, Hulo N (2010). PROSITE, a protein domain database for functional characterization and annotation. Nucleic Acids Res.

[68] Fontes M, Teh T, Kobe B (2000). Structural basis of recognition of monopartite and bipartite nuclear localization sequences by mammalian importin-alpha. J Mol Biol.

[69] Zhao T (2000). Heterochromatin protein 1 binds to nucleosomes and DNA in vitro. J Biol Chem.

[70] Lomberk G, Bensi D, Fernandez-Zapico ME, Urrutia R (2006). Evidence for the existence of an HP1-mediated subcode within the histone code. Nat Cell Biol.

[71] Spassov V, Yan L (2008). A fast and accurate computational approach to protein ionization. Protein Sci.

[72] Sampath S (2007). Methylation of a histone mimic within the histone methyltransferase G9a regulates protein complex assembly. Mol Cell.

[73] Papamokos G (2012). Structural role of RKS motifs in chromatin interactions: a molecular dynamics study of HP1 bound to a variably modified histone tail. Biophys J.

[74] Jacobs S, Khoransanizadeh S (2002). Structure of HP1 chromodomain bound to a lysine 9-methylated histone H3 tail. Science.

[75] Munari F (2013). Structural plasticity in human heterochromatin protein 1β. PLoS ONE.

[76] Edgar R (2004). MUSCLE: multiple sequence alignment with high accuracy and high throughput. Nucleic Acids Res.

