# Supplementary figures and images for: Evidence supporting a critical contribution of intrinsically disordered regions to the biochemical behavior of full-length human HP1γ
Source: J Mol Model. 2015 Dec 17;22:12. doi: 10.1007/s00894-015-2874-z (PMC4683166; doi:10.1007/s00894-015-2874-z)

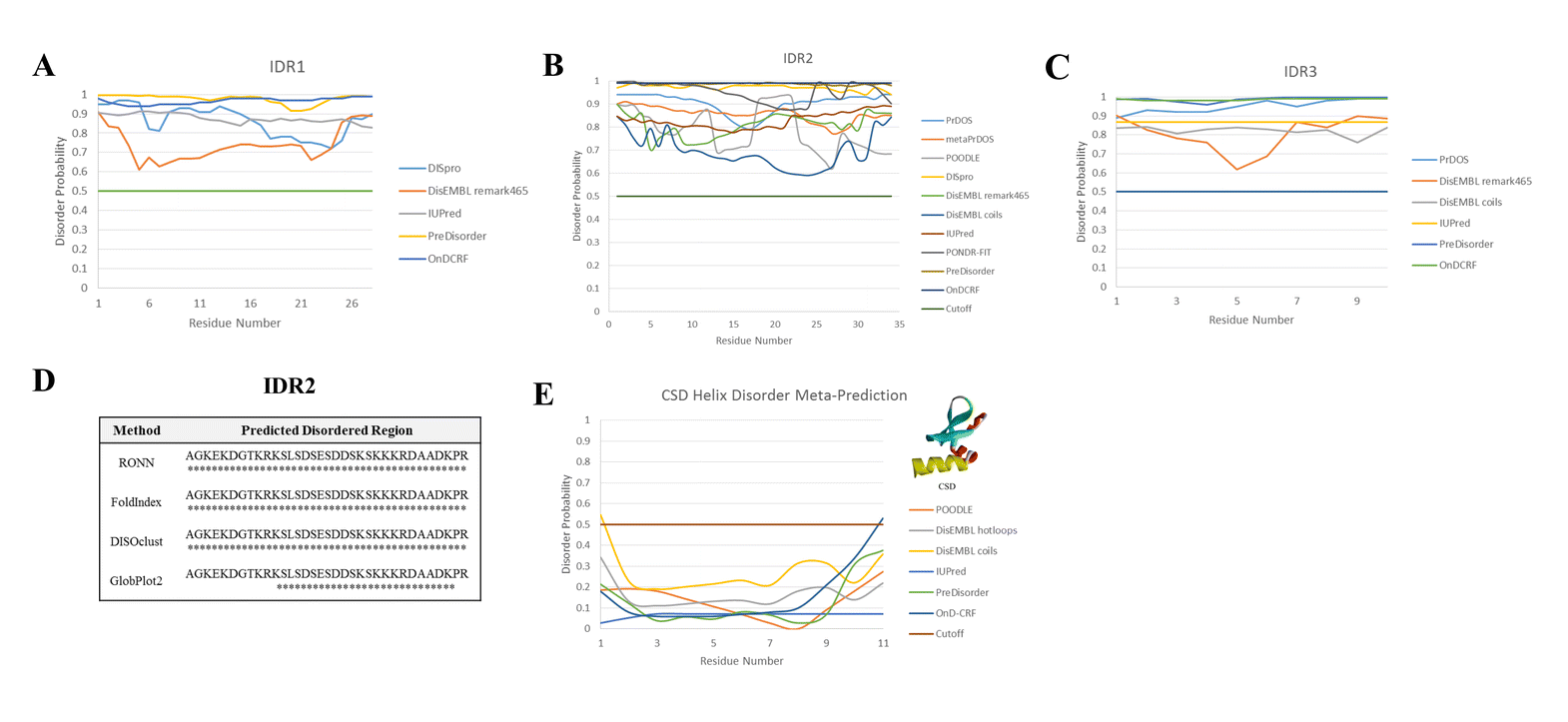

Supplement: Supplementary file 1 — Disorder meta-prediction for IDR1, IDR2, and IDR3. Multiple sequence-based disorder prediction algorithms were used to predict the propensity of the linker region toward disorder in solution. Disorder probability values above the cut-off value of 0.5 are considered to be disordered. d Additional disorder predictions for IDR2 were performed using RONN, FoldIndex, DISOclust, and GlobPlot2. e Additionally, the same disorder meta-prediction was used on a coiled region of the HP1γ chromoshadow domain (PQIVIAFYEER; residue 161-171) as a negative control. The results of this meta-prediction show that most of this region is ordered as opposed to the IDRs. (GIF 83 kb) [file 894_2015_2874_Fig12_ESM.gif]

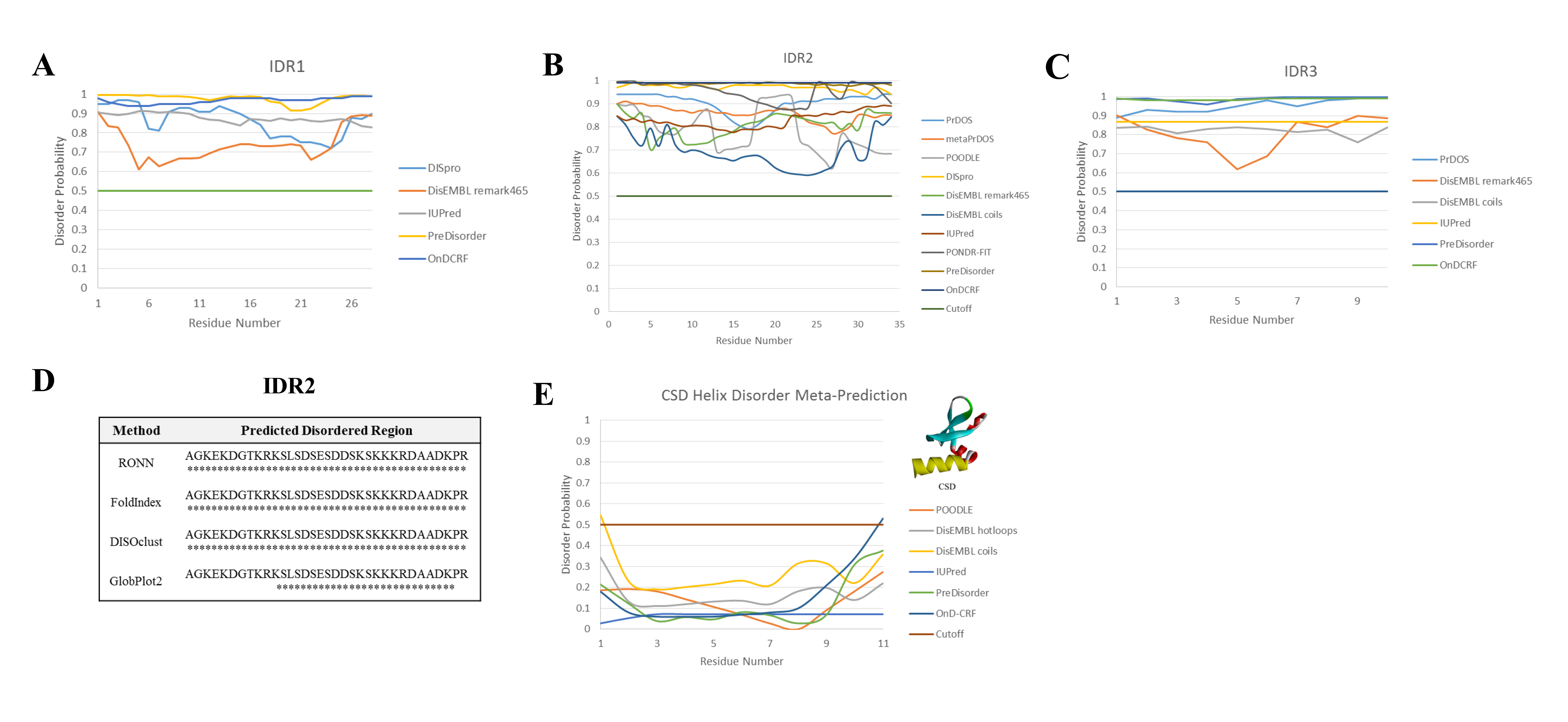

Supplement: Supplementary file 2 — High Resolution (TIFF 2772 kb) [file 894_2015_2874_MOESM1_ESM.tiff]

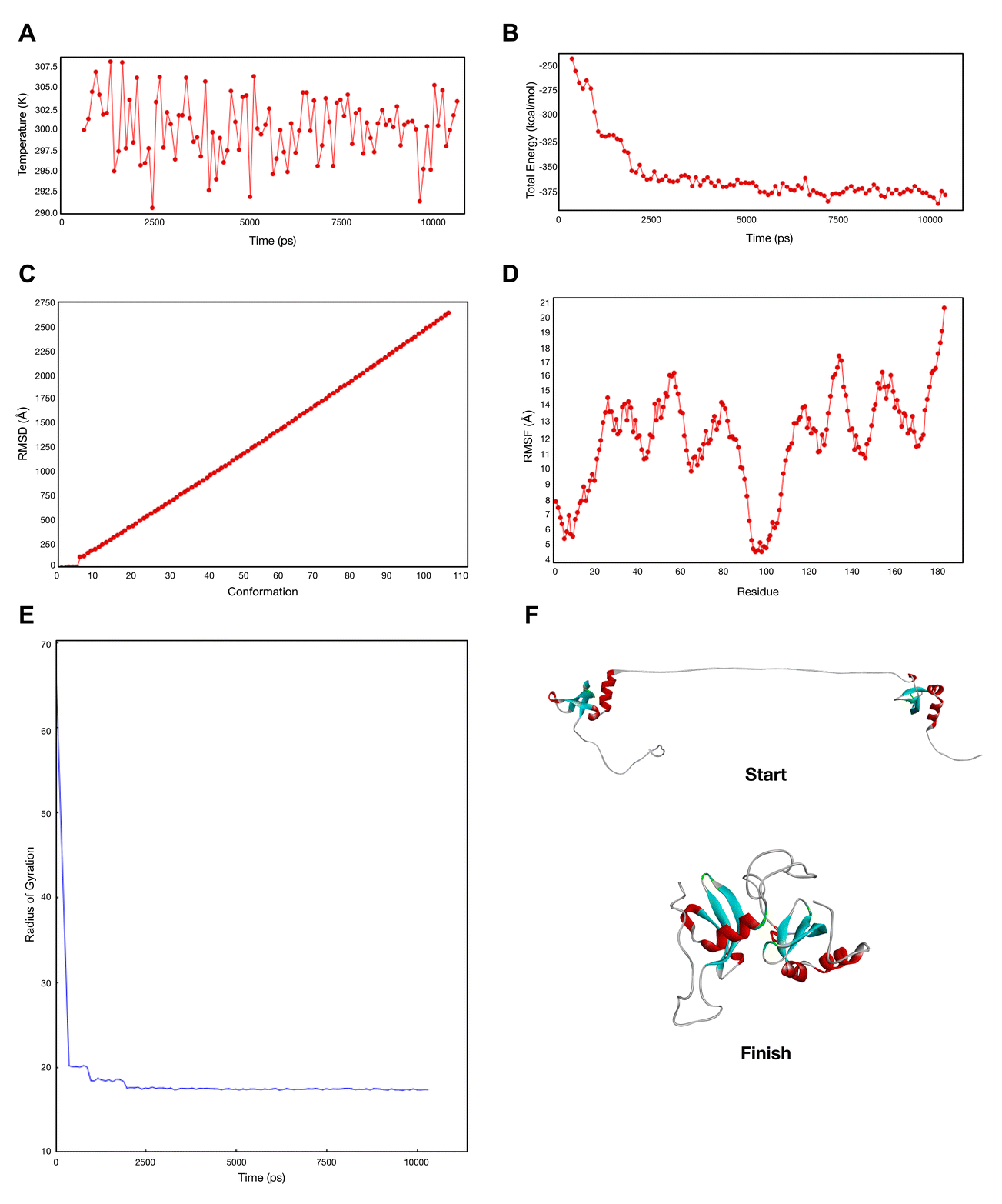

Supplement: Supplementary file 3 — Extended molecular dynamics simulations of the HP1γ monomer. a, b A 10-ns MD simulation was performed on the HP1 monomer with implicit-solvation. The temperature and total energy profiles are represented. c A numerical representation of the flexibility and mobility of this protein during the simulation time was obtained by calculating the root-mean-square deviation (RMSD) and d root-mean-square fluctuation (RMSF). e Radius of gyration calculation for the generalized born (GB) simulation. f Assemblage of conformers obtained during a short 10-ns MD simulation. (GIF 111 kb) [file 894_2015_2874_Fig13_ESM.gif]

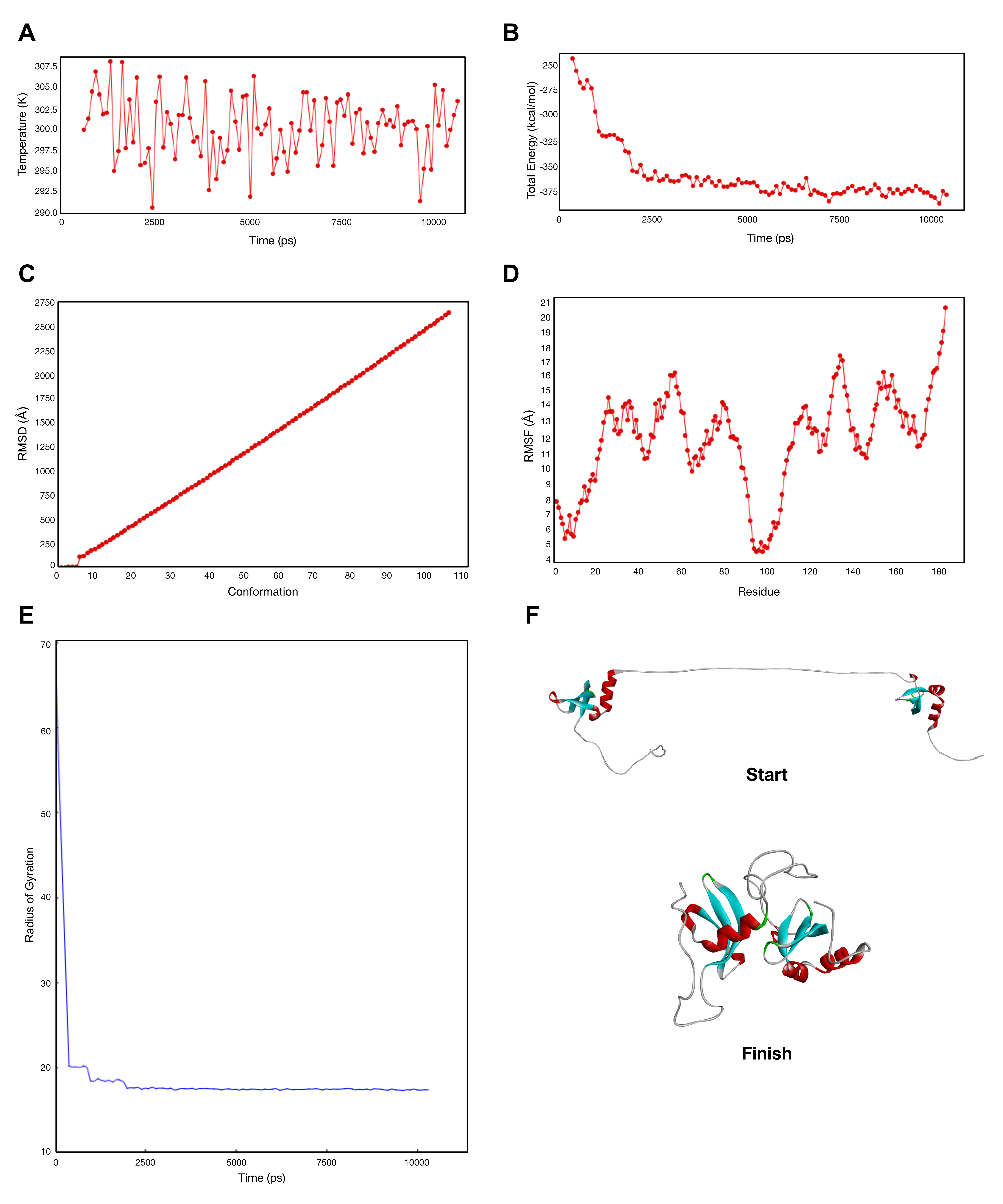

Supplement: Supplementary file 4 — High Resolution (TIFF 4010 kb) [file 894_2015_2874_MOESM2_ESM.tiff]
