# Supplementary material for: Evidence supporting a critical contribution of intrinsically disordered regions to the biochemical behavior of full-length human HP1γ
Source: J Mol Model. 2015 Dec 17;22:12. doi: 10.1007/s00894-015-2874-z (PMC4683166; doi:10.1007/s00894-015-2874-z)
Supplement: Supplementary file 10 — (DOC 22 kb) [file 894_2015_2874_MOESM8_ESM.doc]

**Supplemental Text 1**

Example Calculation of Linear Motif Analysis Meta-Score: Phosphorylation of position S-93: Predicted to be phosphorylated by NetPhosk 1.0, NetPhos 2.0, Kinasephos 2, DIPHOS, PhosphoSVM, Scansite, and PPSP (7 out of the 8 programs used; Table S2a). Meta-score = 7/8 or 0.875. Maximum score = 1 if all programs predict PTLM at that residue.
